# Atypical hemolytic uremic syndrome in a patient with thalassemia and a CFH gene mutation: a case report

**DOI:** 10.3389/fmed.2026.1659141

**Published:** 2026-04-27

**Authors:** Jing Wu, Lu Yang, Shanshan Hu, Lu Liu, Xia Yang, Jing Yuan, Yan Zha

**Affiliations:** Department of Nephrology, NHC Key Laboratory of Pulmonary Immunological Disease, Guizhou Provincial Key Laboratory of Pathogenesis and Prevention of Common Chronic Diseases Research, Guizhou Provincial People's Hospital, Guiyang, China

**Keywords:** atypical hemolytic syndrome, case report, CFH, eculizumab, thalassemia

## Abstract

Atypical hemolytic uremic syndrome (aHUS) is a complement-associated thrombotic microangiopathy (TMA). Thalassemia is a hemolytic anemia triggered by defects in the beta-globin gene. Mutations in the complement factor H (CFH) gene are associated with the development of aHUS; however, CFH gene mutations coexisting with thalassemia have rarely been reported. CFH gene mutations are susceptibility factors that can trigger complement overactivation during pregnancy. When aHUS is combined with thalassemia, large amounts of hemoglobin are released from fragmented erythrocytes, leading to amplification of the complement cascade and resulting in a “two-hit” mechanism that may affect the efficacy of C5 inhibitors. Here, we report a case of pregnancy-associated aHUS in a patient with thalassemia and a CFH gene mutation. After admission to the hospital, plasmapheresis and hemodialysis were used to treat TMA. Following confirmation of aHUS, eculizumab was initiated immediately, and the erythroid maturation agent luspatercept was added for the treatment of thalassemia. The patient was weaned off hemodialysis, and there was no recurrence of aHUS during the follow-up period. This diagnostic and therapeutic approach may provide a new strategy for the use of C5 inhibitors to treat patients with aHUS and thalassemia.

## Introduction

Atypical hemolytic uremic syndrome (aHUS) is a thrombotic microangiopathy (TMA) caused by dysregulation of the complement pathway. aHUS is characterized by microangiopathic hemolytic anemia, thrombocytopenia, and renal dysfunction (1–3). aHUS is associated with 50–60% of patients having genetic abnormalities associated with the complement activation pathway (1). In patients with aHUS who have genetic mutations, the disease often requires a first and a second hit to develop (4). aHUS can be triggered by infections, pregnancy, and other factors; some mutation carriers with combined triggers do not develop the disease. Therefore, there may be a threshold of stress that the body can tolerate, in which an initial trigger (e.g., a genetic mutation) combined with a “two-hit” mechanism triggers disease onset when an additional stress is present (e.g., pregnancy) (5).

In cases of aHUS with a complement factor H (CFH) gene mutation, the prognosis is poor, and the disease often progresses to end-stage renal disease without appropriate treatment (6). With the introduction of anti-C5 therapy (humanized monoclonal antibodies that block C5 activation), improved therapeutic options are available for patients with aHUS (7). Because aHUS is a rare critical disease with a genetic predisposition, accurate and comprehensive genetic analysis is an essential element of clinical routine, supporting diagnosis, guiding clinical management and decision-making, enabling appropriate assessment of recurrence risk, and determining when to stop treatment and the subsequent duration of follow-up. In addition, it is critical to properly inform at-risk relatives about counseling and testing (7).

Thalassemia is a chronic hemolytic anemia caused by an imbalance in globin chain synthesis due to an abnormality in one or more beta-globin genes, resulting in changes in the structure and function of hemoglobin. The most common types are beta- and alpha-thalassemia (8). Complement is a powerful innate immune defense mechanism; however, its overactivation can lead to host tissue damage (9). Broken erythrocytes release large amounts of hemoglobin, which can directly bind to initiating factors of the alternate pathway (e.g., C3b) and promote the formation of C3 convertase. This process amplifies the complement cascade. Hemoglobin can bind to lectins or other pattern-recognition molecules, initiating complement activation through the lectin pathway. The resulting oxidative stress or cellular damage may release autoantigens, which then trigger the formation of antigen–antibody complexes. These complexes indirectly activate the classical pathway. Together, these mechanisms stimulate complement activation through multiple pathways and may reduce the efficacy of C5 inhibitors (5, 10–12).

Here, we report a case of aHUS with a mutation in the CFH gene; notably, the patient had coexisting thalassemia, and that patient’s offspring were also found to have aHUS, with a mutation at the same locus.

## Case presentation

A 30-year-old woman underwent a cesarean section at another hospital 2 weeks ago. On postoperative day 3, renal function tests showed a serum creatinine level of 179 μmol/L, hemoglobin of 77 g/L, and a platelet count of 61 × 10^9^/L, along with tea-colored urine. Thrombotic microangiopathy was suspected, and plasmapheresis was performed three times; however, serum creatinine progressively increased while hemoglobin and platelet levels progressively declined. The patient was admitted to our department for further consultation 3 days prior. The patient had chest tightness and shortness of breath after activity, accompanied by low urine output, fatigue, dizziness, occasional cough, and sputum, producing a small amount of white mucous sputum that was easily expectorated. She was found to have *β*-thalassemia half a year before, and her mother also had the disease. She was admitted to the hospital with a blood pressure of 168/102 mmHg, showing an anemic appearance, with pale eyelids, lips, and nail beds; scattered petechiae were on both upper limbs; her lungs had slightly coarse respiratory sounds, and slight crackles were heard; her abdomen was flat and soft, with a 4-cm transverse post-cesarean section wound, which was already scabbed over; a deep venous catheter was in the right groin, which was secured in place, and her wound dressing was dry without any obvious oozing of blood or fluids. There was mild symmetrical depressed edema in both lower limbs. The relevant examinations after admission are shown (Table 1).

**Table 1:** Experimental inspection results.

Items	Value of a number	Normal range	Units
Hb	58	114–163	g/L
PLT	51	125–350	*10^9/L
Fragmented red blood cells	1.4	<1	%
Ret	4.8	0.5–1.5	%
LDH	449	120–250	U/L
Cr	447	41–73	umol/L
eGFR	11	≥90	ml^−1^ ▪min^−1^ ▪ (1.73 m^2^)^−1^
Albumin	30.4	40–55	g/L
24-h urine protein quantification	4.74	<0.3	g/d
sC5b-9	541	75–219	ng/ml
H-factor	509.8	246.60–417.69	ng/ml

Three days after the cesarean section, the patient showed progressive elevation in serum creatinine, accompanied by a progressive decrease in hemoglobin and platelets. After admission, the reticulocyte count and lactate dehydrogenase, total bilirubin, and indirect bilirubin levels were all elevated, and fragmented red blood cells were >1%. Considering thrombotic microangiopathy, hemodialysis and plasma exchange were performed immediately to remove excess toxins and pathogenic antibodies. At the same time, ADAMTS13 activity was normal, and its inhibitory antibody was negative, which excluded thrombotic thrombocytopenic purpura. The patient did not present with gastrointestinal symptoms such as abdominal pain, diarrhea, or abdominal distension, and stool routine and stool culture were normal; therefore, typical hemolytic uremic syndrome was excluded. Given these findings, atypical hemolytic uremic syndrome was considered. Further testing of the soluble membrane attack complex, complement H factor, B factor, I factor, H factor antibody, and CH50 was performed. The results showed sC5b-9 of 541 ng/mL and complement H factor 509.04 ng/mL, supporting the diagnosis of aHUS.

**Figure 1:**
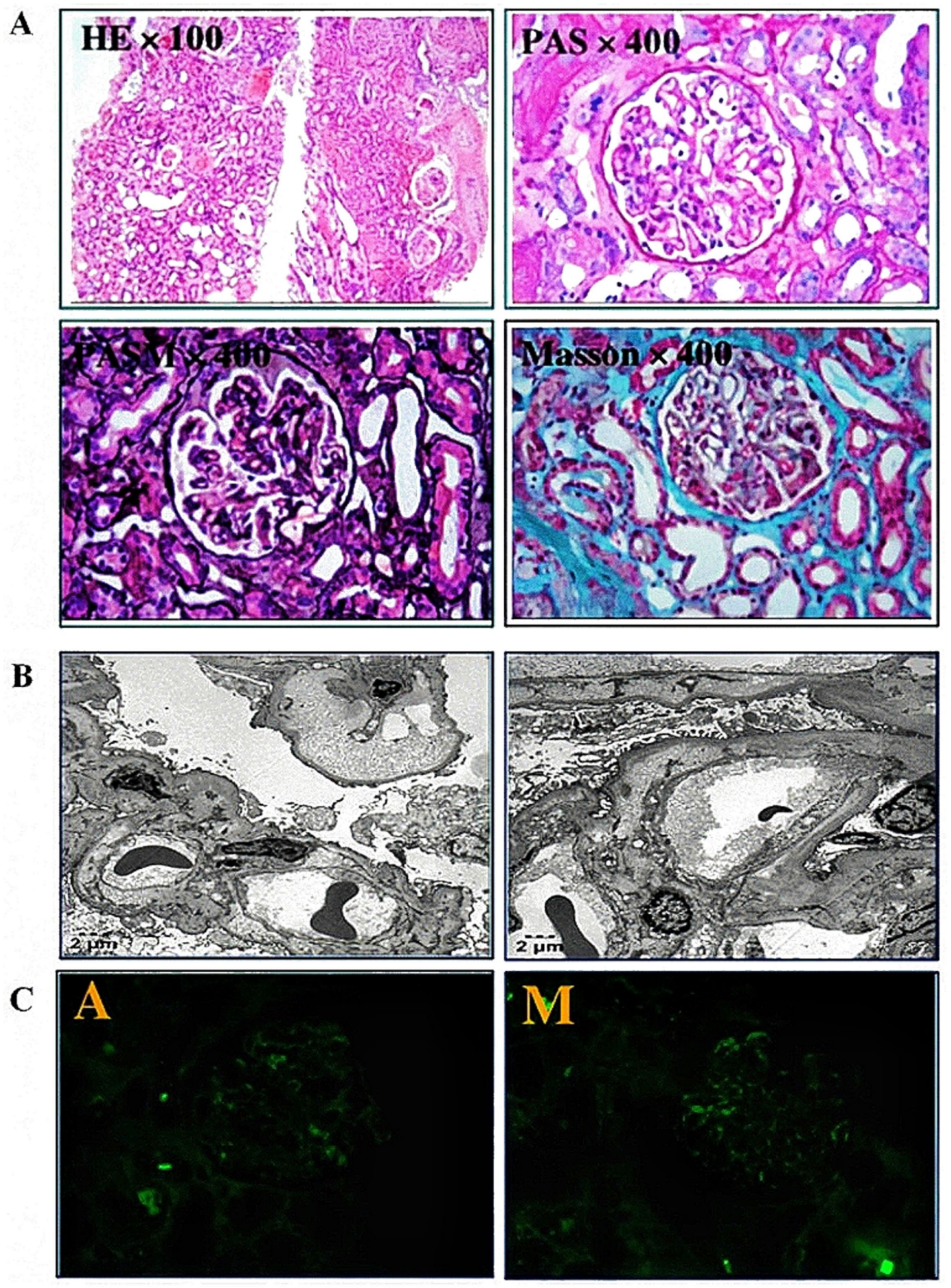
Pathological picture of renal biopsy. **(A)** Light microscopy showing an increased number of glomerular endothelial cells, compression and occlusion of capillary loops, thickening of basement membranes, renal interstitial edema, and focal inflammatory cell infiltration with fibrosis. **(B)** Electron microscopy showed segmental endothelial cell hyperplasia, compression of capillary loops, and marked widening of the subendothelial gap in the capillary wall, within which flocculent material was observed. **(C)** Immunofluorescence did not show clear immune complex deposition.

Through a literature review, several national and international guidelines/consensus recommended complement C5 inhibitors as the first-line treatment for aHUS, and with piperacillin tazobactam for infection prophylaxis as adjunctive prophylaxis, eculizumab 900 mg once weekly was initiated immediately and then changed to 1,200 mg once every 2 weeks for injection after 4 weeks. During plasma exchange, 600 mg was supplemented after each plasma exchange to maintain stable eculizumab blood concentrations. The patient was up-to-date on meningitis vaccination after puerperium. Because most aHUS cases are caused by complement gene defects, to further clarify the etiology of aHUS, guide treatment, and predict prognosis, genetic testing was performed, and the results suggested the CFH chr1q31.3 heterozygous deletion (suspected pathogenic variant). Plasma exchange (eight times in total) treatment was stopped after the diagnosis was further clarified. After 4 weeks of active eculizumab treatment, the patient’s platelets rose to and stabilized in the normal range, reticulocytes and lactate dehydrogenase gradually decreased to normal, blood albumin rose to normal, and the quantitative 24-h urinary protein decreased to 0.29 g/d. Under luspatercept treatment, hemoglobin fluctuated between 50 and 70 g/L, and intermittent transfusion therapy was still required. The patient’s renal function indices did not show a significant decrease, and serum creatinine fluctuated between 447 and 557umol/L. In order to clarify whether the cause of renal insufficiency was combined with primary renal disease, renal biopsy was performed when the patient passed the puerperium, and the pathological results were consistent with renal damage of thrombotic microangiopathy (Figure 1), which further clarified the diagnosis histologically.

**Figure 2:**
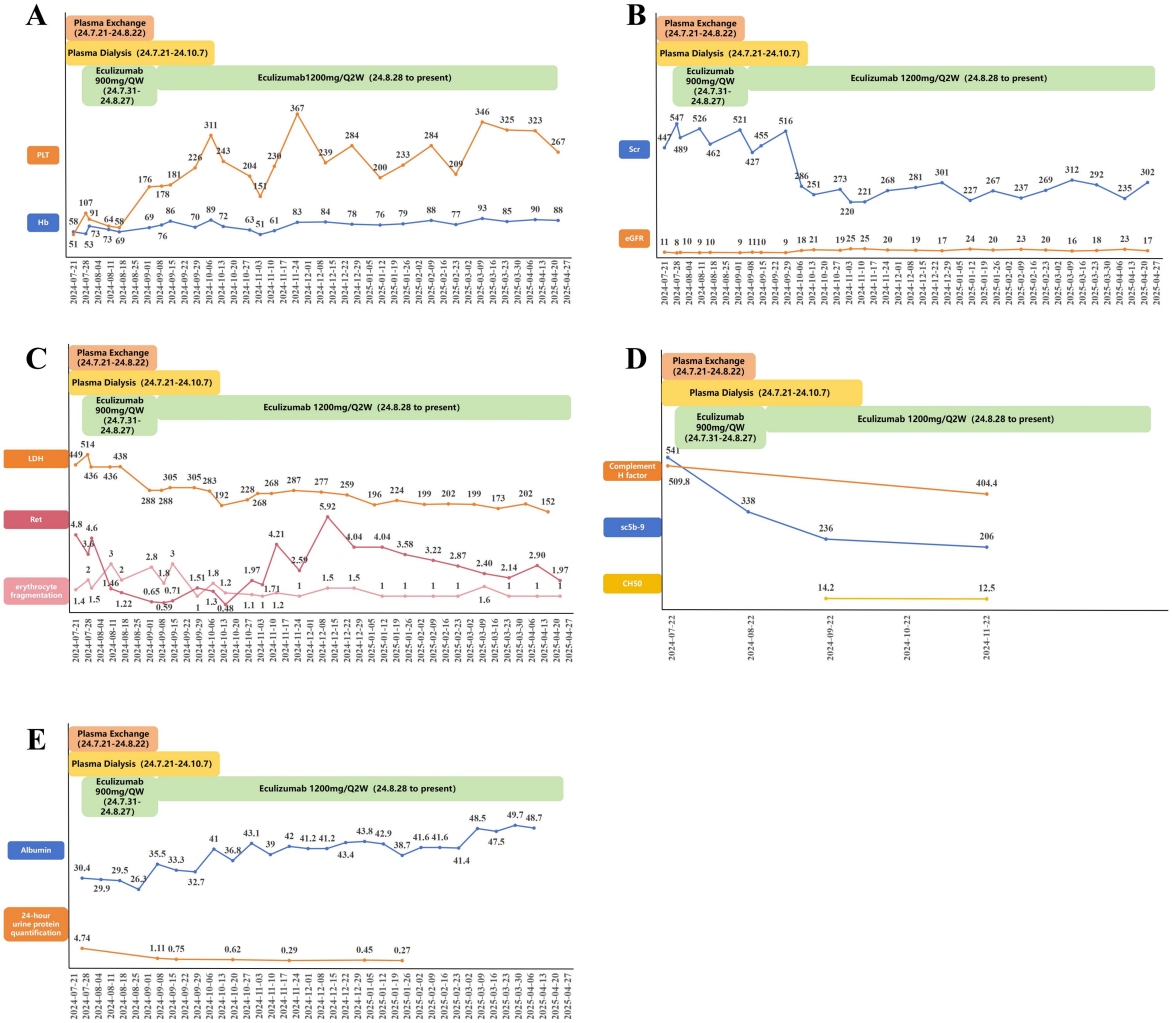
Patient follow-up. **(A)** Hemoglobin and platelet indices; **(B)** serum creatinine and estimated glomerular filtration rate indices; **(C)** lactate dehydrogenase, fragmented erythrocyte, and reticulocyte indices; **(D)** soluble membrane attack complexes, total complement levels, and factor H indices of complement; and **(E)** albumin and quantitative 24-h urine protein indices.

**Figure 3:**
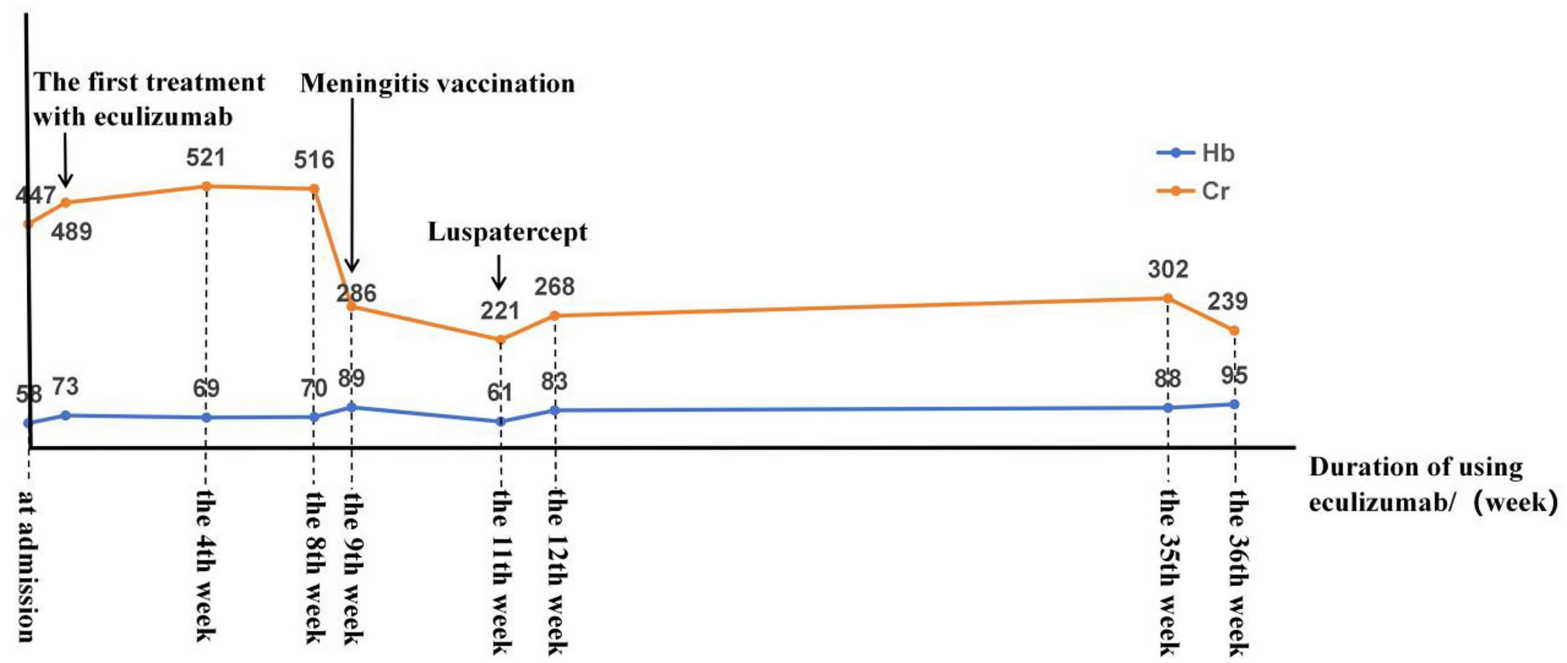
Patient treatment. After admission, the patient underwent various tests and was diagnosed with aHUS. The main treatment was eculizumab, supplemented by luspatercept, hemodialysis, and plasma exchange for symptomatic treatment.

During the period of renal function indicators, when admitted to our department, the patient had a serum creatinine level of 447 μmol/L, which then increased to 547 μmol/L. After 4 weeks of treatment with eculizumab, serum creatinine did not decrease significantly, but the patient’s serum creatinine in the 9th week decreased slightly compared with the previous week, and in the 10th week, serum creatinine dropped to 273 umol/L, hemodialysis was stopped, and the quantitative amount of 24-h urinary protein was also decreased from the initial 4.74 g/d to 0.27 g/d. Simultaneously, the levels of the main pathogenic factors, sC5b-9 and factor H, decreased, with sC5b-9 being reduced to the normal range. The total complement CH50 level can be used to monitor the efficacy of eculizumab and the decrease in its effectiveness. Platelets can be stabilized within the normal range after 1 month of regular use of eculizumab. All disease-related treatments seemed to be on the upswing, but unfortunately, the patient’s hemoglobin level and fragmented red blood cells did not stabilize after 15 weeks of eculizumab treatment. However, we added luspatercept, an erythroid maturation agent, to eculizumab treatment at week 16, and since then, the patient’s hemoglobin level has been maintained at 80–93 g/L, and the fragmented red blood cells have been consistently less than 1% (Figure 2). The body eliminates encapsulated bacteria, and complement C5 plays a crucial role in this process. Eculizumab, as a C5 inhibitor, may suppress the body’s ability to clear capsulated bacteria to some extent, increasing the risk of infections caused by capsulated bacteria such as *Neisseria meningitidis*. Therefore, meningococcal vaccination is required before initiating complement inhibitor therapy. Given the specific circumstances of the patient, piperacillin was administered for infection control during the initial phase. Vaccination will be scheduled promptly after the postpartum period (Figure 3).

## Family history

**Figure 4:**
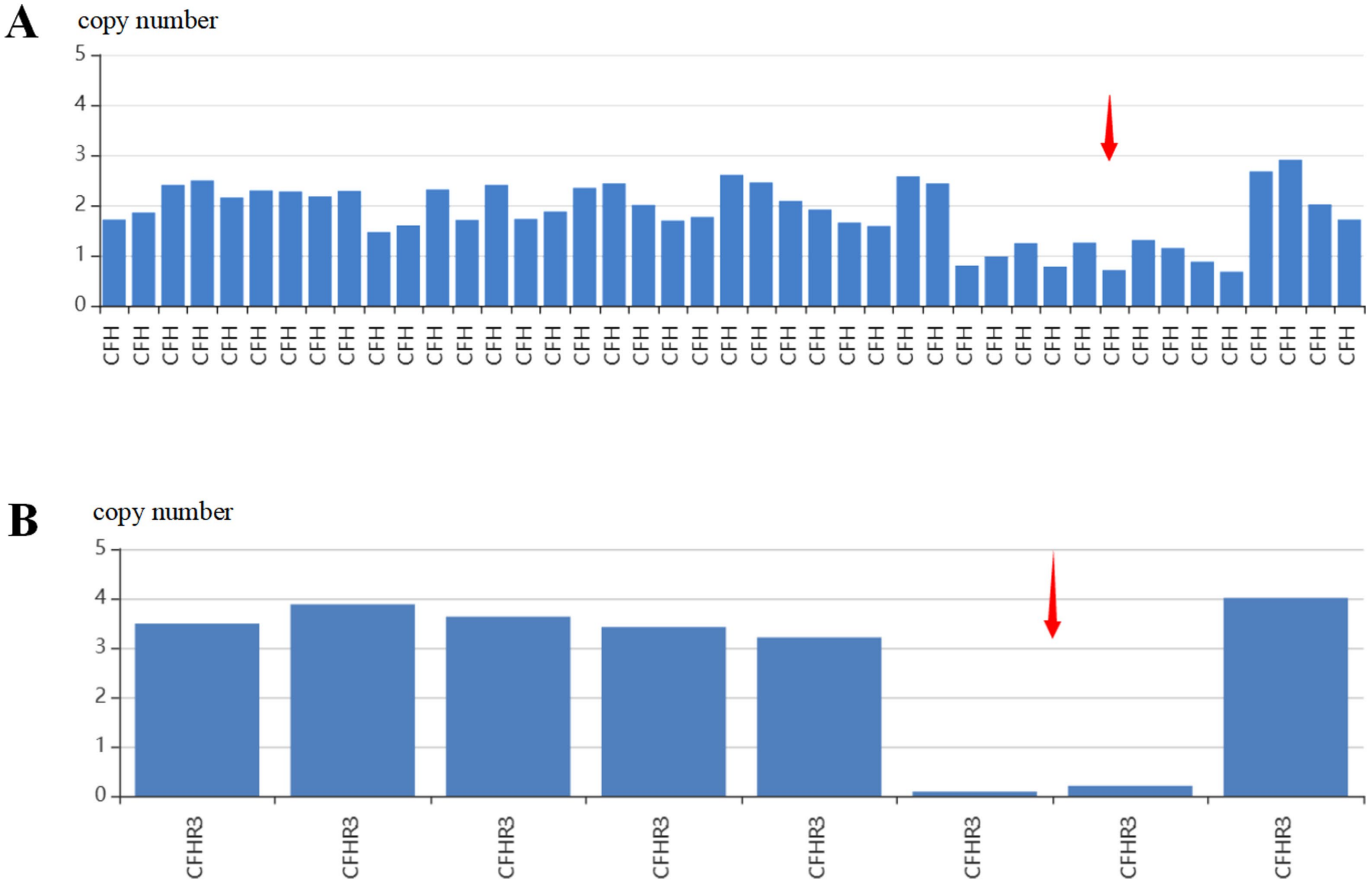
Genetic testing of the patient’s offspring. **(A)** Heterozygous deletion of the CFH gene exon 16–20. **(B)** Pure heterozygous deletion of exon 5 of CFHR3.

The father of this patient (proband) was previously healthy, and the mother had thalassemia with no history of TMA on either side. However, the father’s offspring presented with bilateral lower extremity edema at 6 months of age, and a detailed history revealed an upper respiratory tract infection 1 week prior to the onset of the disease. Post-admission tests showed hemoglobin 66 g/L ↓, platelets 168 × 10^9^/L ↓, reticulocyte count 0.37 × 10^12^/L ↑, fragmented erythrocytes 2.5% ↑, creatinine 131 μmol/L ↑, estimated glomerular filtration rate 77 mL.min^−1^ ▪ (1.73 m^2^)^−1^ ↓, and quantitative 24-h urinary protein 1.69 g/d ↑. In view of the above laboratory tests, TMA was considered, and we performed testing of complement pathway-related factors: factor H antibody 410.13 ng/mL ↓ (reference range: 474.38 ng/mL-1346.75 ng/mL), soluble complement membrane attack complex (sC5b-9) 595 ng/mL ↑ (reference range: 75 ng/mL-219 ng/mL), ADAMTS13 activity: 41.69% ↓ (reference range: 42.16–126.37%), negative inhibitory antibodies, and normal concentrations of complement factors H, B, and I. Considering the proband as a CFH gene mutation, we likewise tested the genes of the father’s offspring, and the results returned: heterozygous deletion of exons 16–20 of the CFH gene, heterozygous deletion of exon 5 of the CFHR3 gene, and heterozygous carriage of the HBB c.52A > T (Figure 4).

**Figure 5:**
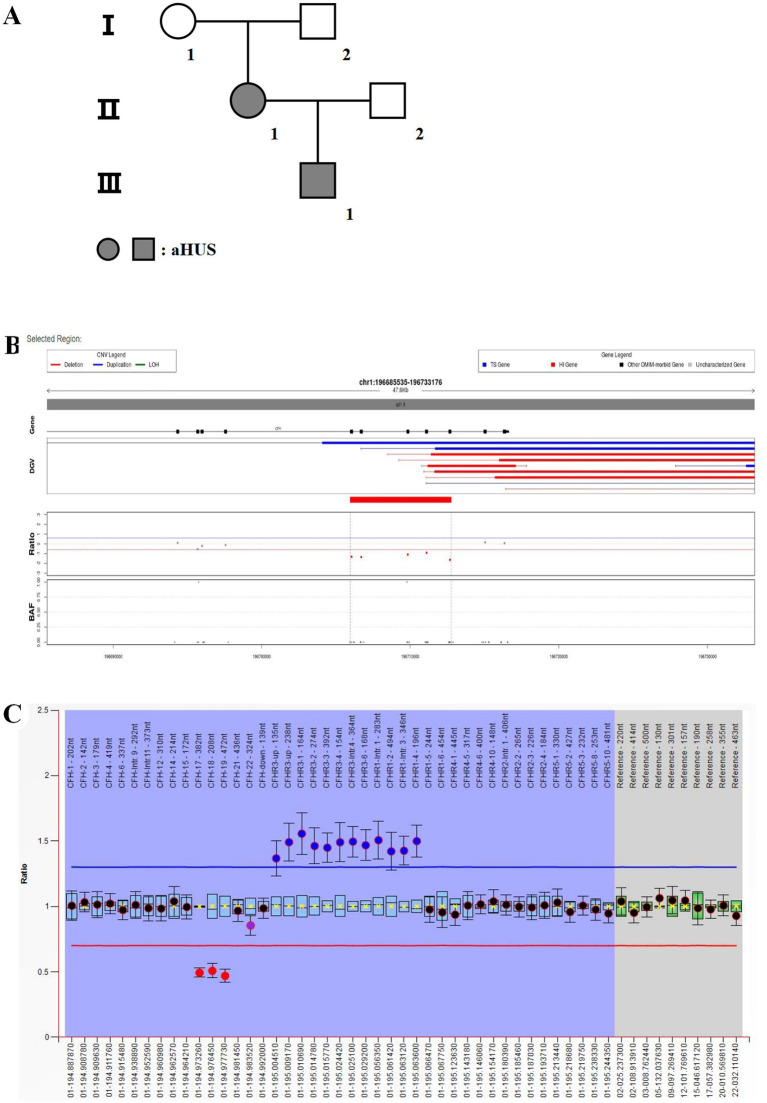
Genetic testing of the patient’s family. **(A)** Family genealogy. Two members of this family (II-1 and III-1) have aHUS. I-1: Mother; I-2: father; II-1: proband; II-2: spouse; III-1: offspring. **(B)** A heterozygous deletion variant in the region of chromosome 1q31.3 was considered as a deletion of exons 16–20 and its neighboring introns of the *CFH* gene (NM_000186.4). **(C)** MLPA analysis of the prior (II-2): Heterozygous deletion mutations in exon 17–19 of the *CFH* gene, increased gene copy number detected in intron 4, exon 6, exon 1–4 and upstream regions of the *CFHR3* gene, and presence of duplicated mutations, and increased gene copy number detected in exon 2, exon 4, intron 1, and intron 3 of the *CFHR1* gene. Copy number increases were detected in the *CFHR1* gene exon 2, exon 4, intron 1, and intron 3, and duplications were detected.

Because both the proband and her offspring had mutations with large deletions, in order to further clarify the inheritance process of the proband family tree, we analyzed the CFH and CFHR genes of her parents and partner by multiplexed ligation-dependent probe amplification (MLPA) and did not find any abnormalities in the exon copy number of the genes in question (Figure 5). From this, we concluded that the proband gene was a *de novo* mutation and that the father’s offspring inherited the mutated gene. Based on the patient’s treatment experience, we immediately administered eculizumab to her offspring at the beginning of the disease, and the patient’s offspring recovered well in terms of blood and renal function.

## Discussion

aHUS is a rare and critical disease, belonging to one type of thrombotic microangiopathy (TMA), with an incidence rate of approximately 2 per million adults and 3.3 per million children (13), and an overall prevalence rate of about 7 per million (1, 14). The clinical manifestations of TMA mainly include microangiopathic hemolytic anemia (MAHA), thrombocytopenia, and multi-organ damage in the kidneys, brain, eyes, heart, gastrointestinal system, and other organs, whereas aHUS is most prominently characterized by kidney involvement (1). It is currently believed that the main mechanism of aHUS pathogenesis is abnormal activation of the alternate bypass pathway and endothelial damage due to triggers such as infection, pregnancy, and surgery, as a second blow on top of the presence of complement regulatory proteins or genetic variants of complement intrinsic proteins, or the presence of anticomplement H factor (CFH) antibodies. In the era of pre-complement therapy, there is an acute morbidity and mortality rate of 25%, with approximately 50% of patients progressing to end-stage renal disease (ESRD) within 1 year.

Numerous mutants associated with variations in complement factor H (CFH), membrane cofactor protein (MCP/CD46), complement factor I (CFI), complement factor B (CFB), CFH-related genes (CFHR1, CFHR2, CFHR3, CFHR4), and diacylglycerol kinase *ε* (DGKE) genes have been identified, making individuals susceptible to aHUS, and the clinical manifestations and disease severity depend on the pathogenic genetic background (15, 16). CFH is the most important regulator of the complement substitution pathway. Genotypic/phenotypic correlations of genetic variants within CFH may result in a wide range of disorders, depending on where the variants are located in the functional region of CFH. Mutations in the CFH gene are one of the most common causes of aHUS, accounting for approximately 20–30% of cases, with a high tendency to relapse and a poor prognosis (17). A multicenter cohort study showed that more than one aHUS-related or anti-CFH gene mutation was confirmed in 45% of 851 patients (45% children, 55% adults) (18); therefore, early identification and diagnosis are extremely important for aHUS patients with CFH gene mutations (19). Most CFH gene mutations are heterozygous variants, and plasma levels of factor H and complement C3 can be normal in patients, making improved genetic testing essential. The response to plasma therapy and long-term prognosis of different aHUS patients are variable, with some patients having plasma dependence or resistance accompanied by an increased risk of complications such as infection and thrombosis (2). In aHUS patients with mutations in the CFH gene, 30–50% of patients relapse even after receiving plasma therapy, and 70–80% develop ESRD after 5 years (20). The introduction of complement inhibitors, such as eculizumab, has greatly improved the prognosis of patients with aHUS. In this case, hemodialysis and plasma exchange were initiated immediately after the initial diagnosis of TMA; however, the patient’s blood picture and renal function did not improve significantly until the genetic test results returned a mutation in the CFH gene, which led to a definitive diagnosis of aHUS and brought about a turnaround in the patient’s treatment.

With the increase of medical evidence, several domestic and international guidelines or consensus have recommended eculizumab as the first-line therapeutic agent for aHUS (16, 21–23). The results of C08-002, the core phase II clinical study of eculizumab, showed that the patients’ complement activity was rapidly inhibited within 1 h after the first administration of the drug, and this inhibitory effect lasted for 2 years. Follow-up results showed that 76% of patients achieved complete remission of TMA during the 2-year treatment period (24). Another core study, C10-004, noted a significant increase in platelet counts in patients after 7 days of eculizumab treatment (25). In 98% (40/41) of the 41 patients, platelet counts returned to normal after a median of 8 days (range, 0–84 days) (26). In addition, in adult aHUS patients, the improvement in renal function with eculizumab also took effect at 1 week (26). Further studies have found that early initiation of eculizumab therapy can assist 80–83% of patients to be successfully weaned off dialysis after 26 weeks of treatment with additional renal benefit (26). Therefore, once aHUS is diagnosed, treatment with eculizumab should be initiated as early as possible (27), especially for aHUS patients who develop mutations in the complement factor gene, which increases the risk of recurrence by 16.2-fold compared to patients in whom no variants are detected (28), and there is a correlation between the risk of recurrence in aHUS and the expression of the aHUS genotype (2). aHUS patients in whom, depending on individualization, there may be CFH, C3, CFB, and CFI mutations, CFH gene rearrangements, anti-CFH autoantibodies, or genetic polymorphisms associated with mutations. Patients with a history of prior aHUS, a family history of aHUS, who have received a kidney transplant, and who have severe extrarenal manifestations are at a high risk for recurrence (29). In particular, patients with aHUS in the presence of mutations in the complement H factor gene are at higher risk of recurrence and therefore should be treated with eculizumab earlier (24, 30, 31). For special populations, such as pregnant women, eculizumab has also shown a favorable safety profile. In a real-world study, no increased risk of adverse outcomes or fetal abnormalities was found in pregnant women exposed to eculizumab, and the rate of miscarriage was consistent with that in the general population (32). There has been experience with eculizumab in more than 400 pregnant patients (33), with no increased risk of fetal malformations or fetal-neonatal toxicity (34).

Considering the complex etiology of aHUS and the different prognoses of different pathogenic factors, the duration of eculizumab treatment remains debatable. The KDIGO guideline points out (35) that all patients with complement-associated aHUS should be treated for at least 6–12 months, and those with a high risk of recurrence should be treated for more than 12 months or even need lifelong treatment. The timing of discontinuation should be carefully determined based on patient-specific circumstances (including genetic variants, transplantation history, relapse history, and dialysis history), and the risk of relapse after discontinuation should be comprehensively assessed (24). It has been noted (24) that regular monitoring of blood counts, renal function, and urine protein after 1, 2, 3, 6, 9, and 12 months of discontinuation should be considered for relapse when serum creatinine is greater than the upper limit for the same age or increases by 15% of baseline, proteinuria increases by 25%, platelet counts are less than 150 × 10^9^/L, or there is evidence of hemolysis. In the event of recurrence, restarting C5 inhibitor therapy within 24–48 h is recommended. If there are more than two recurrences of aHUS, lifelong treatment should be considered. A multicenter study of eculizumab for the treatment of aHUS (36) included 93 patients with aHUS from 5 clinical trials and followed them for 6 years. Results showed a 93% reduction in the incidence of TMA symptoms during eculizumab treatment. In terms of improving renal function, eGFR remained stable in eculizumab-treated patients during up to 6 years of follow-up. Among the patients who never discontinued the drug, only 3.9% required dialysis at the last follow-up. However, among patients who discontinued the drug, renal function showed a decreasing trend with time since discontinuation, and the percentage of patients requiring dialysis was 14.3%. This further suggests that long-term treatment with eculizumab is associated with improvement and maintenance of stable renal function, whereas discontinuation of eculizumab is associated with a trend of progressive decline in renal function over time and a greater likelihood of recurrence after discontinuation in patients with high-risk factors for recurrence, such as genetic mutations (36).

From the patient’s follow-up, platelets stabilized in the normal range in the 4th week of eculizumab treatment, hemodialysis was stopped in the 10th week, and sC5b-9 decreased to normal in the 15th week, which is in line with the recovery of the indices reported in the above literature. In particular, the overall recovery of the patient’s indices was slower after the use of eculizumab, and it was considered that this might be related to coexisting diseases affecting the efficacy of eculizumab in this patient. The patient had aHUS with a CFH gene mutation combined with thalassemia. It was previously suggested that the patient was diagnosed with thalassemia 6 months ago, and his hemoglobin could be stabilized at 100 + g/L. After the onset of aHUS, the patient’s hemoglobin level showed a progressive decline. This implies that aHUS and thalassemia seem to be positive feedback mechanisms. As there was no obvious causative factor in the early stage, the genetic mutation of aHUS with thalassemia acted as a blow to bring the patient’s disease risk pressure to the threshold range but did not cause the patient to develop clinical manifestations associated with the disease. It is not until after pregnancy that the whole body, including the complement system, receives an “immune storm” as a second blow, triggering the development of aHUS and anemia. When we inhibit the relevant complement pathway with a C5 inhibitor (eculizumab), the large amount of hemoglobin produced by hemolytic anemia positively feeds back to keep the complement system active. The patient’s hemoglobin level was not significantly elevated by the previous treatment with eculizumab and is now maintained at 80 + g/L after treatment with luspatercept. sC5b-9 in the plasma of patients with *β*-thalassemia has been shown to be strongly correlated with the severity score of thalassemia (37). The soluble complement membrane attack complex sC5b-9 is elevated in thalassemia (25). Hemolytic disorders are often associated with dysregulation and hyperactivation of the complement system (8, 38, 39), which may be induced by free extracellular hemoglobin (5, 10–12). *In vitro* studies have demonstrated that hemoglobin-mediated activation of the alternative complement pathway involves two key mechanisms: hemoglobin released from lysed red blood cells not only directly activates the pathway but also binds to C3, thereby enhancing its cleavage capacity and triggering a cascade amplification effect. Compared with endothelial cells in normal human serum, aHUS patients exhibit significantly higher C3 deposition in endothelial cells under identical hemoglobin concentrations. This phenomenon is particularly pronounced in patients with genetic mutations (such as H or C3 gene mutations) (5). It has been reported that heme binds preferentially to intact C3 and C3a, but not to C3 degradation fragments C3b or C3d (9). Molecular docking predicts that heme is in close proximity to the thioester bond, which is critical for C3 activation. In addition, *in vitro* experiments have shown that exposure of human C3 to heme results in enhanced C3(H2O) production and isotype interactions (5). These effects may contribute to the activation of the complement system in the blood. However, disease associations and pathogenic pathway targets specifically related to aHUS and thalassemia have not yet been identified. Considering the patient’s pre-existing genetic background of *β*-thalassemia, this progressive decrease in hemoglobin level after the onset of aHUS suggests that the possibility of a common direct or indirect molecular pathway between the two disorders contributing to the development of anemia remains to be confirmed by subsequent studies. Studies have indicated that when healthy individuals exposed to the same concentration of heme are co-cultured with serum from aHUS patients, the deposition of C3 fragments on the surface of endothelial cells from aHUS patients is significantly higher than that observed in serum samples from healthy individuals. Furthermore, higher heme concentrations are correlated with increased C3 deposition on endothelial cell surfaces. This phenomenon is more pronounced in cases involving genetic abnormalities of the complement components (9).

When the patient was followed up for 6 months, unfortunately, the father’s offspring was also admitted to the hospital with symptoms of bilateral lower extremity edema. Based on the diagnosis and treatment of this patient, we immediately performed the genetic testing of the offspring at the early stage of admission, and the results suggested a high degree of overlap with the deletion of the CFH gene in the father’s mother, which, in combination with the clinical manifestations and laboratory tests, supported the diagnosis of aHUS. CFH is a single-chain glycoprotein that inhibits the production of C3 convertase and accelerates the degradation of C3 convertase through competitive binding to C3b and acts as a cofactor for CFI during the cleavage process of C3b (36). Specifically, it interacts with various polyanions (e.g., heparan sulfate and salicylic acid) to develop a greater affinity for C3b, resulting in more effective inactivation of C3b. Endothelial cells and glomerular basement membranes are enriched in polyanion molecules, preventing uncontrolled deposition of C3b on their surfaces (36). According to various studies, mutant proteins have a low binding affinity for both polyanions and C3b on the surface of endothelial cells, which determines their weak control over the complement system (36, 40, 41), leading to aberrant complement activation. The gene encoding CFH and CFH-related (CFHR) proteins is located on the long arm of chromosome 1 (1q32) and has a high degree of homology, which favors genomic rearrangement and copy number variation (CNV) (36). This leads to the deletion of CFHR1, CFHR3, or CFHR4 and the formation of hybrid genes such as CFH-CFHR1. Some of these recombinant or hybrid genes are associated with the production of CFH autoantibodies or the malfunction of hybrid proteins (36). The results of a retrospective study analyzing CFH-CFHR copy number variation (CNV) have been reported in the literature (42), emphasizing the prevalent role of CFH gene defects in the pathogenesis of aHUS and suggesting that abnormalities in CFHR proteins may also play a role. In addition, it has been found that the CFH gene mutation correlation is associated with poor prognosis in patients with aHUS, emphasizing the importance of analyzing and characterizing CFH variants to ensure a complete functional understanding of CFH gene variants. In the present case, the patient was tested for CFH gene fragment deletion, and his parents had normal genetic results, which presumably led to a *de novo* mutation. The deletion fragment at this locus was also inherited by the offspring. This gene is a susceptibility gene, which is considered a “two-hit mechanism” for the development of the disease in the offspring. When an upper respiratory tract infection strikes, it acts as a trigger similar to pregnancy, resulting in the clinical manifestation of aHUS in offspring. Interestingly, second-generation sequencing of the offspring of the patient not only showed mutations in the aHUS-related genes but also showed that the patient was a carrier of the *β*-thalassemia gene. At this time, probably because of the low number of fragmented erythrocytes in the body at the early stage of the onset of the disease in the offspring, as well as the timeliness of our administration of eculizumab, the failure of hemoglobin to rise for a prolonged period after the administration of the drug did not occur in the course of the treatment, suggesting once again that the interconnection between aHUS and thalassemia may affect the therapeutic effect of C5 complement inhibitors.

In conclusion, this case highlights the fact that aHUS combined with hemolytic anemia diseases such as thalassemia may be ineffective when treated with eculizumab alone and that concomitant treatment of related disorders may lead to better clinical outcomes, providing a new perspective on the use of C5 inhibitors in the treatment of aHUS.

## Data Availability

The datasets presented in this study can be found in online repositories. The names of the repository/repositories and accession number(s) can be found in the article/Supplementary material.
