# Supplementary material for: Atypical hemolytic uremic syndrome in a patient with thalassemia and a CFH gene mutation: a case report
Source: Front Med (Lausanne). 2026 Apr 27;13:1659141. doi: 10.3389/fmed.2026.1659141 (PMC13159203; doi:10.3389/fmed.2026.1659141)
Supplement: Supplementary file 1 [file Data_Sheet_1.pdf]

Genetic Testing

Sample ID:	25C139637	Name:	Yao Xu	Gender:	Female
Age:	-	Sample Type:	Peripheral blood	Specimen Received:	2025-05-08
Hospital:	GUIZHOU PROVINCIAL PEOPLE'S HOSPITAL			Medical Record Number:	Inpatient Department
Test Name:	MLPA51:Detection of Atypical Hemolytic Uremic Syndrome CFH Region				
Clinical:	-				
Method:	multiplex ligation-dependent probe amplification ,MLPA				

Test Result: The test results indicate that the subject exhibits a heterozygous deletion mutation in exon 17-19 of the CFH gene, as well as increased gene copy numbers detected in intron 4, exon 6, exon 1-4, and upstream regions of the CFHR3 gene,indicating a duplication mutation. Additionally, increased gene copy numbers was identified in exon 2, exon 4, intron 1, and intron 3 of the CFHR1 gene,indicating a duplication mutation. Clinical physicians are advised to conduct comprehensive analysis based on the subject's clinical manifestations and other test results for clinical diagnosis.

The experimental results comparing this sample with normal controls are presented in the figure below:

Fig.

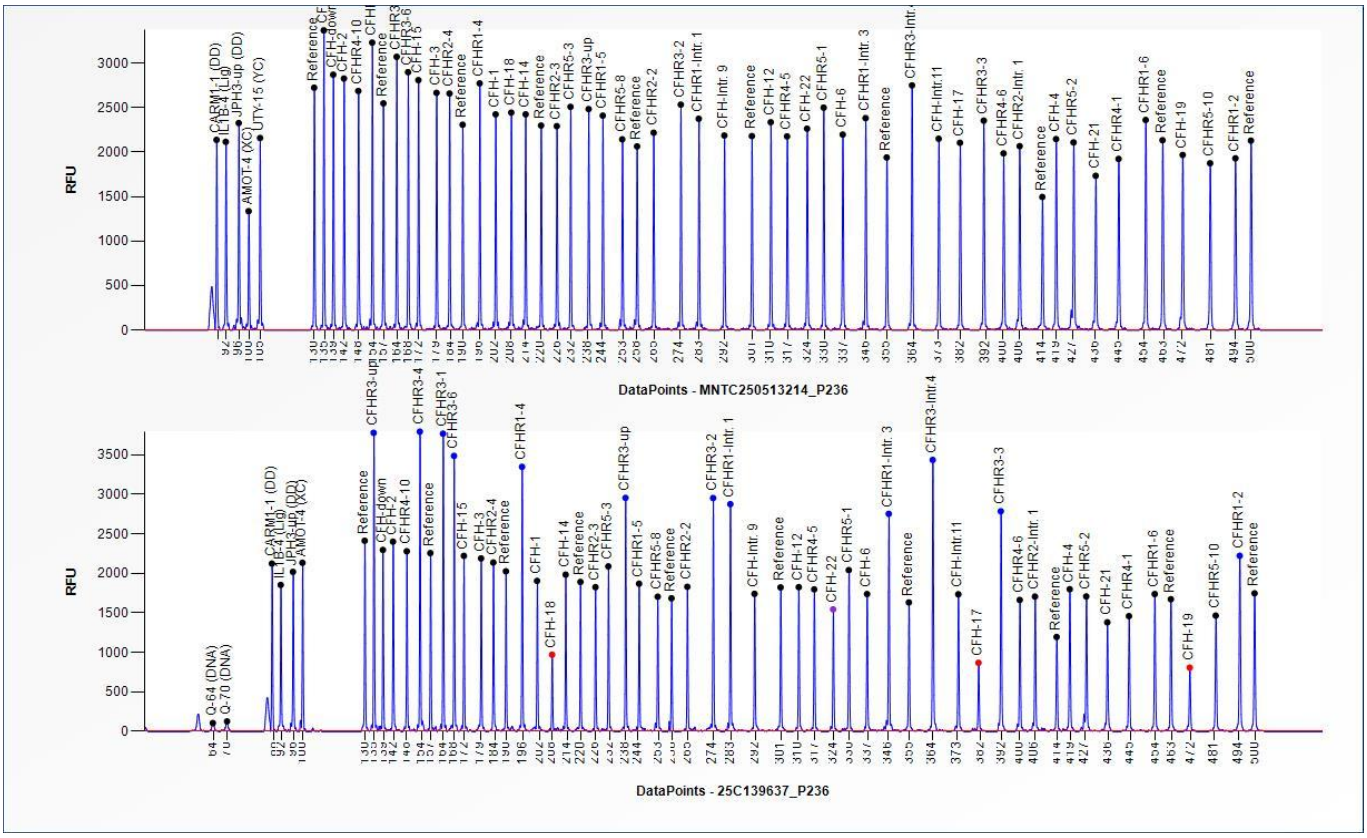

Beijing MyGenostics Medical Laboratory
Adress：No.9 Anqing Street, Juhong Building Block A, Airport Industrial Zone B, Shunyi District, Beijing City.
Tel.：010-87529591/400-003-5255.

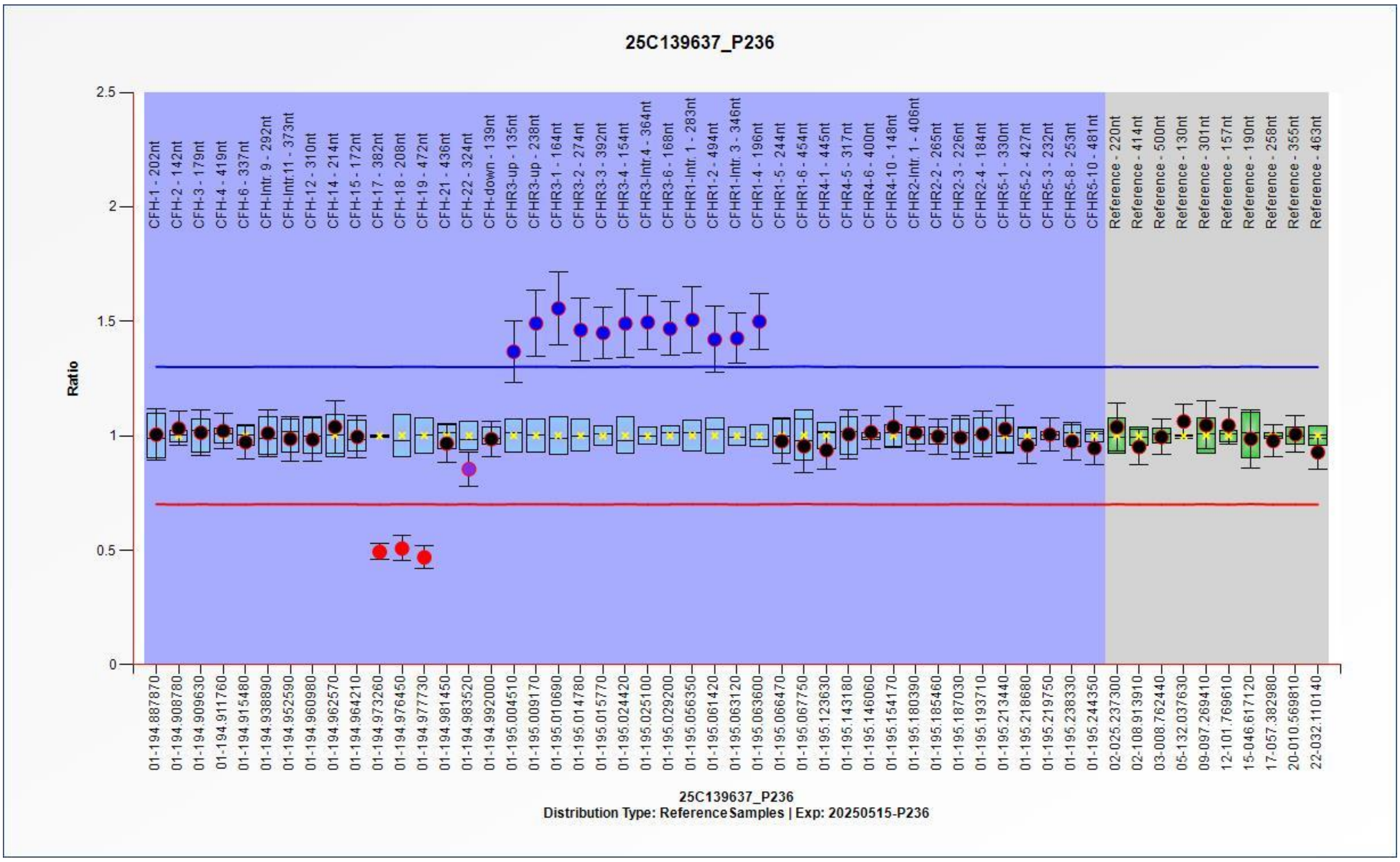

Analysis Note: A fluorescence signal intensity should be between 0.7 and 1.3

Disease Introduction/Genetic significance

Atypical hemolytic uremic syndrome (aHUS) is a clinical syndrome characterized primarily by the triad of microangiopathic hemolytic anemia, acute renal injury, and thrombocytopenia. Unlike typical HUS caused by Shiga toxin-producing Escherichia coli (STEC) infection, the primary pathogenic mechanism in aHUS patients involves abnormal activation of the complement bypass pathway. The incidence of aHUS is approximately 7/1000000. Congenital or acquired abnormal activation of complement pathway is the main pathogenic mechanism of aHUS. Most patients have gene mutations in complement-related factors (CFH, CFI, and CFHR proteins), while some patients are triggered by infections, medications, autoimmune diseases, or inherited cobalamin C metabolic defects.

The CFH gene is a member of the complement activation regulator (RCA) gene cluster, encoding a protein with 20 short consensus repeat (SCR) domains. This protein is secreted into the bloodstream and plays a critical role in complement activation regulation. The complement factor H (CFH) gene family is located on chromosome 1q31.3 and comprises six genes: CFH, CFHR3, CFHR1, CFHR4, CFHR2, and CFHR5. Mutations in this gene are associated with atypical hemolytic uremic syndrome (aHUS) and chronic hypocomplementemic nephropathy. The pathogenic mechanisms primarily include: regulatory region disruption: affecting CFH expression or interactions with other complement factors; gene fusion: forming heterozygous deletions with adjacent genes (such as CFHR1), resulting in aberrant proteins (such as CFH-CFHR1 fusion proteins) that interfere with complement regulatory functions; linkage disequilibrium: linked to specific haplotypes (such as high-risk haplotypes for aHUS). Francis N J et al. found a deletion in the C-terminal region of the CFH gene in a large lineage of atypical hemolytic uremic syndrome (aHUS) in 2012.Microhomologous sequences flanking the breakpoint suggested that this deletion occurred via microhomology-mediated end joining (MMEJ) rather than non-allelic homologous recombination (NAHR). The deletion

led to the formation of a CFH/CFHR3 heterozygous gene, with the N-terminal exon derived from CFH and the C-terminal exon from CFHR3. Functional analysis revealed that although the heterozygous protein exhibits complement regulation activity in liquid phase, it demonstrates defective capacity for complement regulation at the cell surface. Blood. 2008 Feb 1;111(3):1512-4. Epub 2007 Nov15 reported that homozygous deletion of CFHR1/CFHR3 constitutes a risk factor for atypical hemolytic uremic syndrome (HUS). The study elucidated a critical mechanism underlying atypical HUS, namely the production of CFH autoantibodies. These findings suggest that CFHR1/CFHR3 deletion represents a risk factor for CFH autoantibody generation.

Detection range

Probemix	Deletions/Duplications	Point mutation	Methylation	Gene/Region	Exon
P236	Yes	No	No	*CFH*	exon1-4，6，12，14，15，17，18，19，21，22，Intron 9，Intron 11，8.8 kb after exon 22
				CFHR1	exon4-6，2，Intron 1，Intron 3
				CFHR2	exon2，3，4，Intron 1
				CFHR3	exon1-4，6，Upstream，Intron 4
				CFHR4	exon1，5，6，10
				CFHR5	exon1，2，3，8，10

Detection methods and limitations

1. This test utilizes Multiplex Ligation-dependent Probe Amplification (MLPA) technology to analyze the relevant genes.
2. This test cannot detect any changes that lie outside the target sequence of the probes and will not detect copy number neutral inversions or translocations.
3. This test cannot distinguish between triploid and diploid cells in female samples. Furthermore, mRNA quantification requires specially selected samples and is subject to limitations in kinetic curve analysis
4. The interpretation of results at the borderline (critical) value requires further evaluation in conjunction with the clinical context.

Test member: Qing Zhang　　Person Approving: 李淑葵

Analyst: Jing Yang　　Report Date: 2025-05-19

*This report is issued by the entrusted party and/or its affiliated laboratory

*This test is only responsible for the tested sample.

*All the results above are lab data, for Clinical reference only. Furthermore, any genetic testing carries a very small but inherent risk of error.

*The content of the report may not be reproduced, disseminated or excerpted for publication without authorization.

*MyGenostics.Inc owns the final interpretation, if you have any doubts about the report, please contacts us within 5 days.

Beijing MyGenostics Medical Laboratory
Adress：No.9 Anqing Street, Juhong Building Block A, Airport Industrial Zone B, Shunyi District, Beijing City.
Tel.：010-87529591/400-003-5255.

Genetic Testing

Sample ID:	25C139639	Name:	BenYong Xu	Gender:	Male
Age:	-	Sample Type:	Peripheral blood	Specimen Received:	2025-05-08
Hospital:	GUIZHOU PROVINCIAL PEOPLE'S HOSPITAL			Medical Record Number:	-
Test Name:	MLPA51:Detection of Atypical Hemolytic Uremic Syndrome CFH Region				
Clinical:	-				
Method:	multiplex ligation-dependent probe amplification ,MLPA				

Test Result: The current test results indicate that no significant copy number variations were detected in the relevant genes of the subject. The clinical physician is advised to integrate these findings with the subject's clinical presentation and other test results for a comprehensive analysis and clinical diagnosis.

The experimental results comparing this sample with normal controls are presented in the figure below:

Fig.

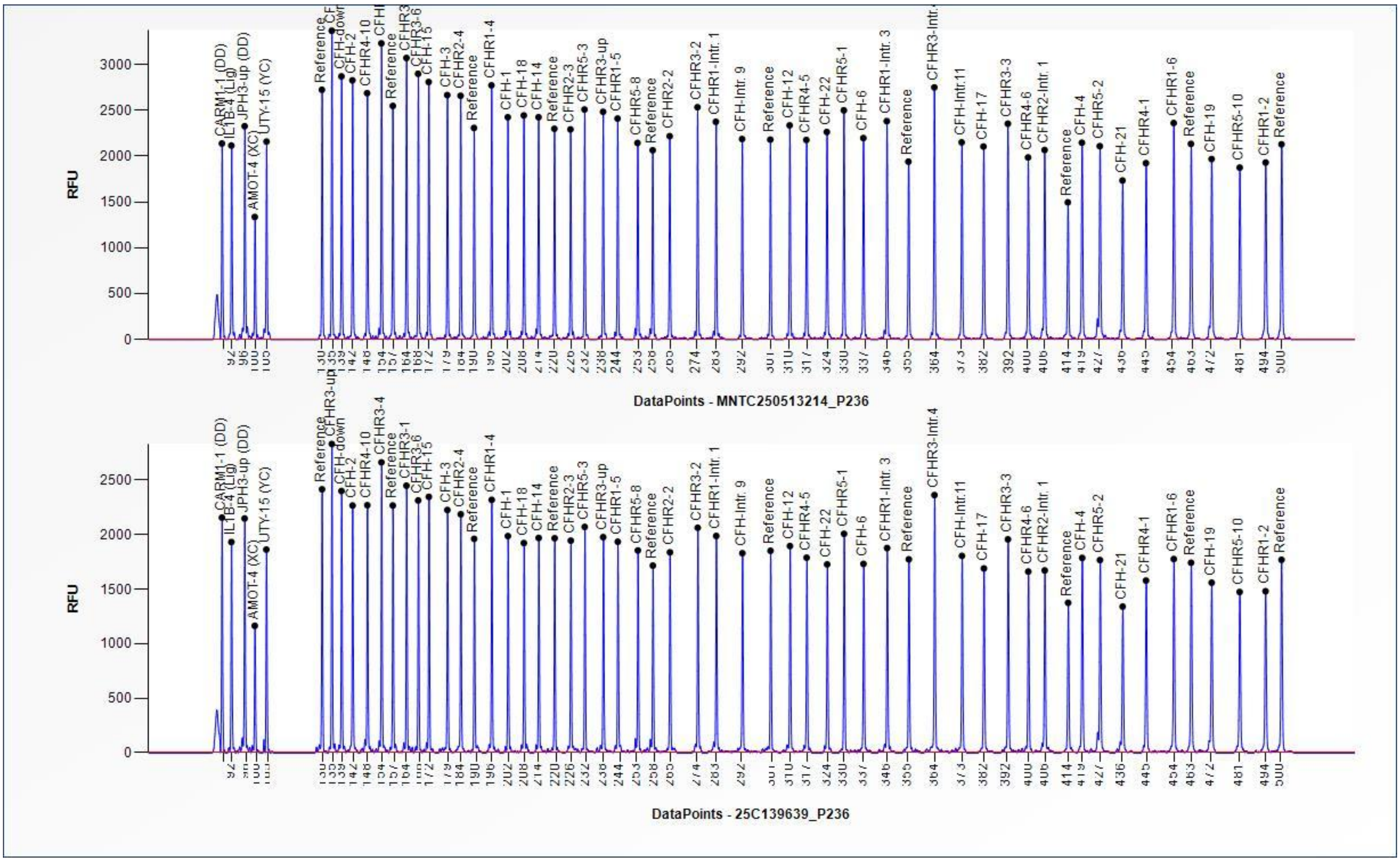

Beijing MyGenostics Medical Laboratory
Adress：No.9 Anqing Street, Juhong Building Block A, Airport Industrial Zone B, Shunyi District, Beijing City.
Tel.：010-87529591/400-003-5255.

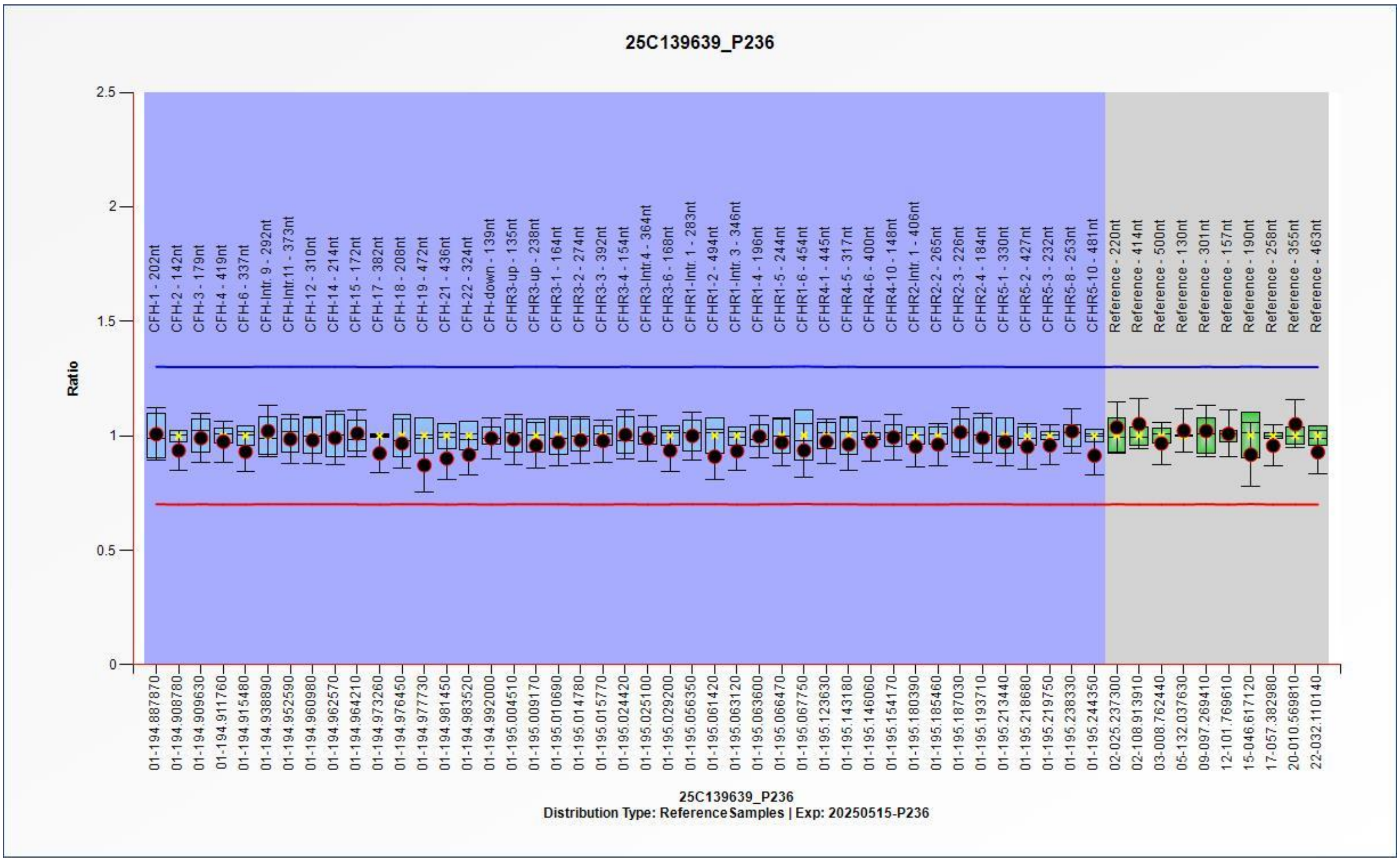

Analysis Note: A fluorescence signal intensity should be between 0.7 and 1.3

Disease Introduction/Genetic significance

Atypical hemolytic uremic syndrome (aHUS) is a clinical syndrome characterized primarily by the triad of microangiopathic hemolytic anemia, acute renal injury, and thrombocytopenia. Unlike typical HUS caused by Shiga toxin-producing Escherichia coli (STEC) infection, the primary pathogenic mechanism in aHUS patients involves abnormal activation of the complement bypass pathway. The incidence of aHUS is approximately 7/1000000. Congenital or acquired abnormal activation of complement pathway is the main pathogenic mechanism of aHUS. Most patients have gene mutations in complement-related factors (CFH, CFI, and CFHR proteins), while some patients are triggered by infections, medications, autoimmune diseases, or inherited cobalamin C metabolic defects.

The CFH gene is a member of the complement activation regulator (RCA) gene cluster, encoding a protein with 20 short consensus repeat (SCR) domains. This protein is secreted into the bloodstream and plays a critical role in complement activation regulation. The complement factor H (CFH) gene family is located on chromosome 1q31.3 and comprises six genes: CFH, CFHR3, CFHR1, CFHR4, CFHR2, and CFHR5. Mutations in this gene are associated with atypical hemolytic uremic syndrome (aHUS) and chronic hypocomplementemic nephropathy. The pathogenic mechanisms primarily include: regulatory region disruption: affecting CFH expression or interactions with other complement factors; gene fusion: forming heterozygous deletions with adjacent genes (such as CFHR1), resulting in aberrant proteins (such as CFH-CFHR1 fusion proteins) that interfere with complement regulatory functions; linkage disequilibrium: linked to specific haplotypes (such as high-risk haplotypes for aHUS). Francis N J et al. found a deletion in the C-terminal region of the CFH gene in a large lineage of atypical hemolytic uremic syndrome (aHUS) in 2012.Microhomologous sequences flanking the breakpoint suggested that this deletion occurred via microhomology-mediated end joining (MMEJ) rather than non-allelic homologous recombination (NAHR). The deletion

led to the formation of a CFH/CFHR3 heterozygous gene, with the N-terminal exon derived from CFH and the C-terminal exon from CFHR3. Functional analysis revealed that although the heterozygous protein exhibits complement regulation activity in liquid phase, it demonstrates defective capacity for complement regulation at the cell surface. Blood. 2008 Feb 1;111(3):1512-4. Epub 2007 Nov15 reported that homozygous deletion of CFHR1/CFHR3 constitutes a risk factor for atypical hemolytic uremic syndrome (HUS). The study elucidated a critical mechanism underlying atypical HUS, namely the production of CFH autoantibodies. These findings suggest that CFHR1/CFHR3 deletion represents a risk factor for CFH autoantibody generation.

Detection range

Probemix	Deletions/Duplications	Point mutation	Methylation	Gene/Region	Exon
P236	Yes	No	No	*CFH*	exon1-4，6，12，14，15，17，18，19，21，22，Intron 9，Intron 11，8.8 kb after exon 22
				CFHR1	exon4-6，2，Intron 1，Intron 3
				CFHR2	exon2，3，4，Intron 1
				CFHR3	exon1-4，6，Upstream，Intron 4
				CFHR4	exon1，5，6，10
				CFHR5	exon1，2，3，8，10

Detection methods and limitations

1. This test utilizes Multiplex Ligation-dependent Probe Amplification (MLPA) technology to analyze the relevant genes.
2. This test cannot detect any changes that lie outside the target sequence of the probes and will not detect copy number neutral inversions or translocations.
3. This test cannot distinguish between triploid and diploid cells in female samples. Furthermore, mRNA quantification requires specially selected samples and is subject to limitations in kinetic curve analysis
4. The interpretation of results at the borderline (critical) value requires further evaluation in conjunction with the clinical context.

Test member:	Qing Zhang	Person Approving:	
Analyst:	Jing Yang	Report Date:	2025-05-19

*This report is issued by the entrusted party and/or its affiliated laboratory

*This test is only responsible for the tested sample.

*All the results above are lab data, for Clinical reference only. Furthermore, any genetic testing carries a very small but inherent risk of error.

*The content of the report may not be reproduced, disseminated or excerpted for publication without authorization.

*MyGenostics.Inc owns the final interpretation, if you have any doubts about the report, please contacts us within 5 days.

Genetic Testing

Sample ID:	25C139640	Name:	Mei Peng	Gender:	Female
Age:	-	Sample Type:	Peripheral blood	Specimen Received:	2025-05-08
Hospital:	GUIZHOU PROVINCIAL PEOPLE'S HOSPITAL			Medical Record Number:	-
Test Name:	MLPA51:Detection of Atypical Hemolytic Uremic Syndrome CFH Region				
Clinical:	-				
Method:	multiplex ligation-dependent probe amplification ,MLPA				

Test Result: The current test results indicate that no significant copy number variations were detected in the relevant genes of the subject. The clinical physician is advised to integrate these findings with the subject's clinical presentation and other test results for a comprehensive analysis and clinical diagnosis.

The experimental results comparing this sample with normal controls are presented in the figure below:

Fig.

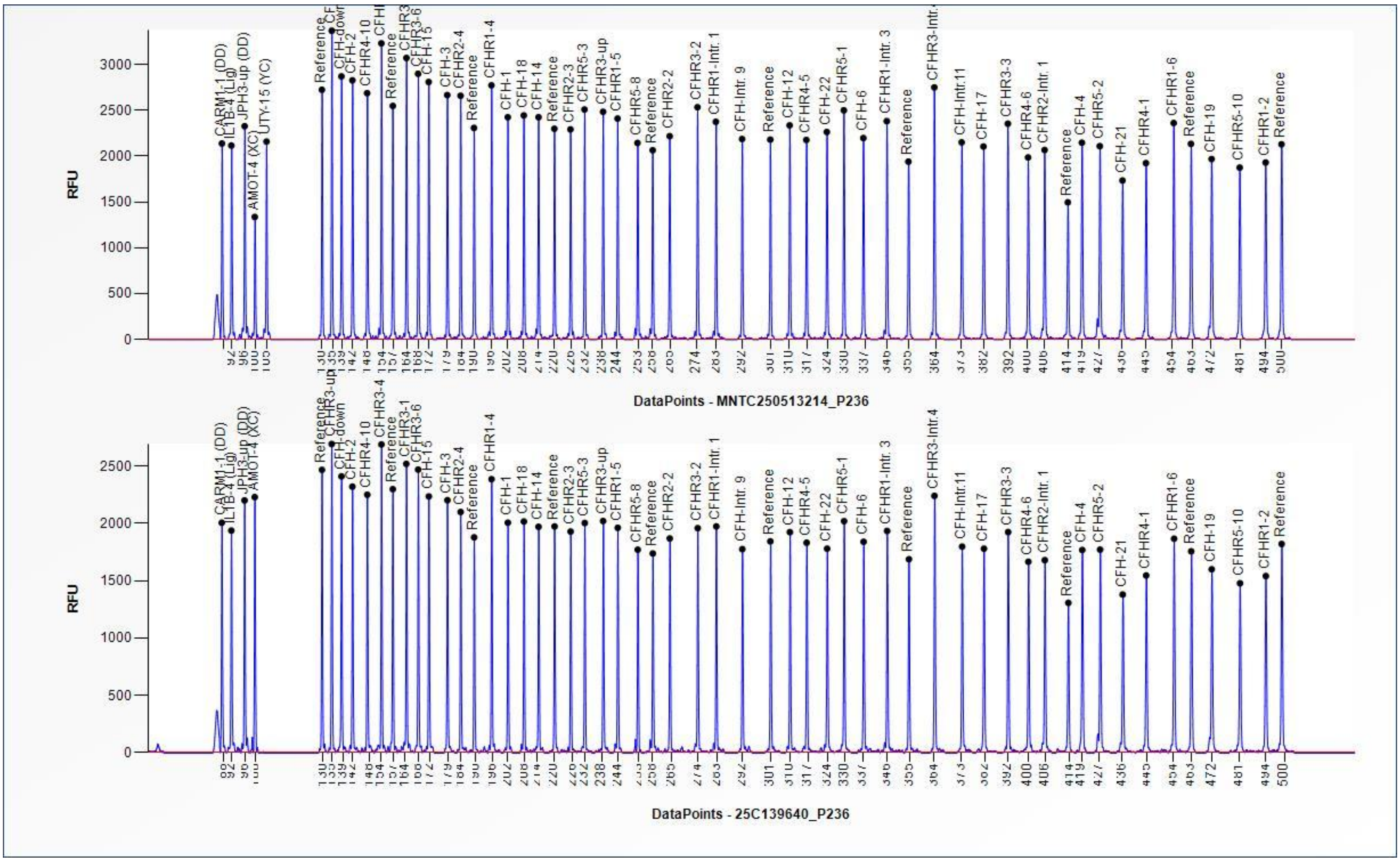

Beijing MyGenostics Medical Laboratory
Adress：No.9 Anqing Street, Juhong Building Block A, Airport Industrial Zone B, Shunyi District, Beijing City.
Tel.：010-87529591/400-003-5255.

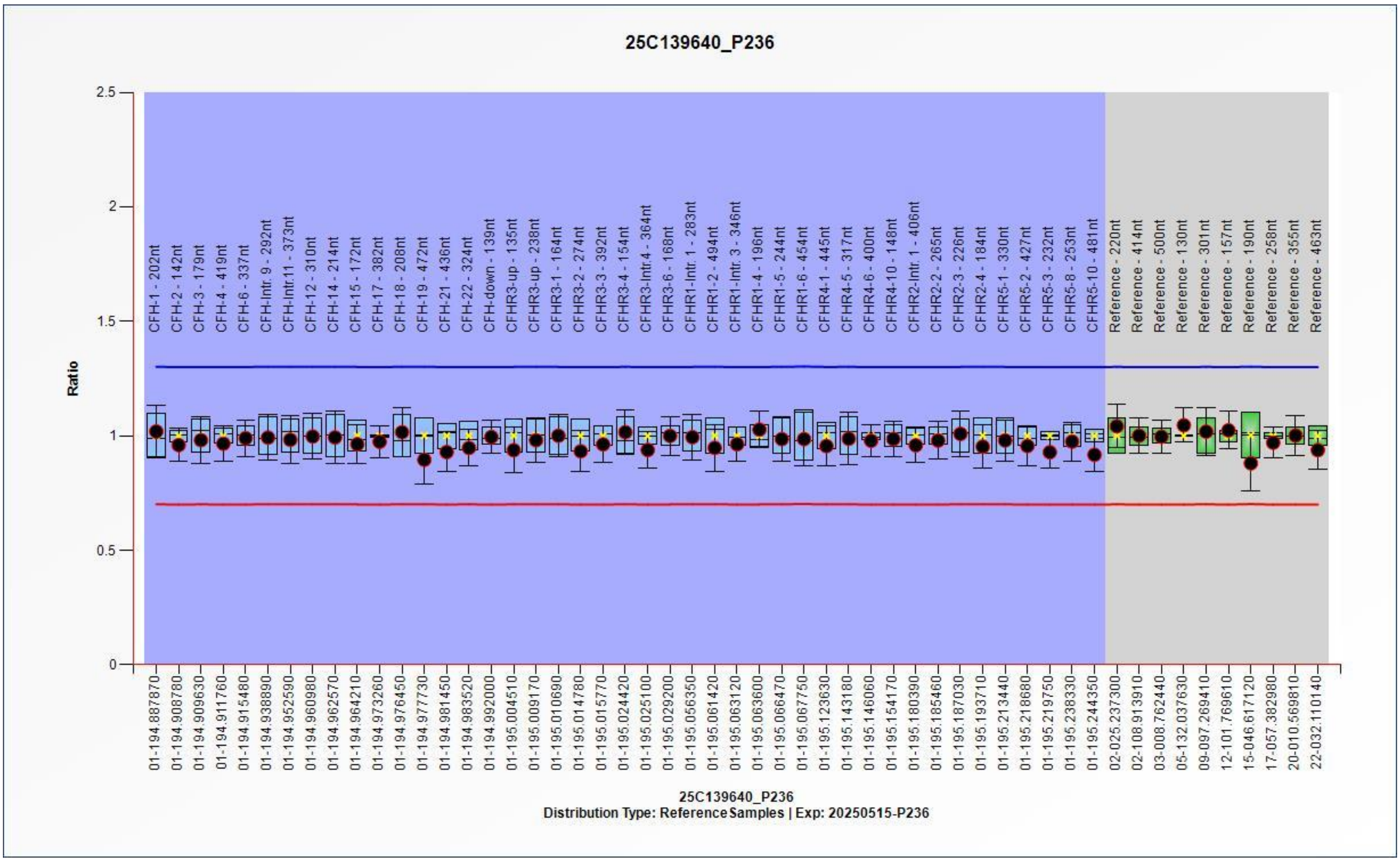

Analysis Note: A fluorescence signal intensity should be between 0.7 and 1.3

Disease Introduction/Genetic significance

Atypical hemolytic uremic syndrome (aHUS) is a clinical syndrome characterized primarily by the triad of microangiopathic hemolytic anemia, acute renal injury, and thrombocytopenia. Unlike typical HUS caused by Shiga toxin-producing Escherichia coli (STEC) infection, the primary pathogenic mechanism in aHUS patients involves abnormal activation of the complement bypass pathway. The incidence of aHUS is approximately 7/1000000. Congenital or acquired abnormal activation of complement pathway is the main pathogenic mechanism of aHUS. Most patients have gene mutations in complement-related factors (CFH, CFI, and CFHR proteins), while some patients are triggered by infections, medications, autoimmune diseases, or inherited cobalamin C metabolic defects.

The CFH gene is a member of the complement activation regulator (RCA) gene cluster, encoding a protein with 20 short consensus repeat (SCR) domains. This protein is secreted into the bloodstream and plays a critical role in complement activation regulation. The complement factor H (CFH) gene family is located on chromosome 1q31.3 and comprises six genes: CFH, CFHR3, CFHR1, CFHR4, CFHR2, and CFHR5. Mutations in this gene are associated with atypical hemolytic uremic syndrome (aHUS) and chronic hypocomplementemic nephropathy. The pathogenic mechanisms primarily include: regulatory region disruption: affecting CFH expression or interactions with other complement factors; gene fusion: forming heterozygous deletions with adjacent genes (such as CFHR1), resulting in aberrant proteins (such as CFH-CFHR1 fusion proteins) that interfere with complement regulatory functions; linkage disequilibrium: linked to specific haplotypes (such as high-risk haplotypes for aHUS). Francis N J et al. found a deletion in the C-terminal region of the CFH gene in a large lineage of atypical hemolytic uremic syndrome (aHUS) in 2012.Microhomologous sequences flanking the breakpoint suggested that this deletion occurred via microhomology-mediated end joining (MMEJ) rather than non-allelic homologous recombination (NAHR). The deletion

led to the formation of a CFH/CFHR3 heterozygous gene, with the N-terminal exon derived from CFH and the C-terminal exon from CFHR3. Functional analysis revealed that although the heterozygous protein exhibits complement regulation activity in liquid phase, it demonstrates defective capacity for complement regulation at the cell surface. Blood. 2008 Feb 1;111(3):1512-4. Epub 2007 Nov15 reported that homozygous deletion of CFHR1/CFHR3 constitutes a risk factor for atypical hemolytic uremic syndrome (HUS). The study elucidated a critical mechanism underlying atypical HUS, namely the production of CFH autoantibodies. These findings suggest that CFHR1/CFHR3 deletion represents a risk factor for CFH autoantibody generation.

Detection range

Probemix	Deletions/Duplications	Point mutation	Methylation	Gene/Region	Exon
P236	Yes	No	No	*CFH*	exon1-4，6，12，14，15，17，18，19，21，22，Intron 9，Intron 11，8.8 kb after exon 22
				CFHR1	exon4-6，2，Intron 1，Intron 3
				CFHR2	exon2，3，4，Intron 1
				CFHR3	exon1-4，6，Upstream，Intron 4
				CFHR4	exon1，5，6，10
				CFHR5	exon1，2，3，8，10

Detection methods and limitations

1. This test utilizes Multiplex Ligation-dependent Probe Amplification (MLPA) technology to analyze the relevant genes.
2. This test cannot detect any changes that lie outside the target sequence of the probes and will not detect copy number neutral inversions or translocations.
3. This test cannot distinguish between triploid and diploid cells in female samples. Furthermore, mRNA quantification requires specially selected samples and is subject to limitations in kinetic curve analysis
4. The interpretation of results at the borderline (critical) value requires further evaluation in conjunction with the clinical context.

Test member: Qing Zhang | Person Approving: 李淑葵

Analyst: Jing Yang | Report Date: 2025-05-19

*This report is issued by the entrusted party and/or its affiliated laboratory

*This test is only responsible for the tested sample.

*All the results above are lab data, for Clinical reference only. Furthermore, any genetic testing carries a very small but inherent risk of error.

*The content of the report may not be reproduced, disseminated or excerpted for publication without authorization.

*MyGenostics.Inc owns the final interpretation, if you have any doubts about the report, please contacts us within 5 days.

Beijing MyGenostics Medical Laboratory
Adress：No.9 Anqing Street, Juhong Building Block A, Airport Industrial Zone B, Shunyi District, Beijing City.
Tel.：010-87529591/400-003-5255.

MGNC25040293_25C139637_Yao Xu

Genetic Testing

Sample ID:	25C139638	Name:	HouJiang Dai	Gender:	Male
Age:	-	Sample Type:	Peripheral blood	Specimen Received:	2025-05-08
Hospital:	GUIZHOU PROVINCIAL PEOPLE'S HOSPITAL			Medical Record Number:	-
Test Name:	MLPA51:Detection of Atypical Hemolytic Uremic Syndrome CFH Region				
Clinical:	-				
Method:	multiplex ligation-dependent probe amplification,MLPA				

Test Result: The current test results indicate that no significant copy number variations were detected in the relevant genes of the subject. The clinical physician is advised to integrate these findings with the subject's clinical presentation and other test results for a comprehensive analysis and clinical diagnosis.

The experimental results comparing this sample with normal controls are presented in the figure below:

Fig.

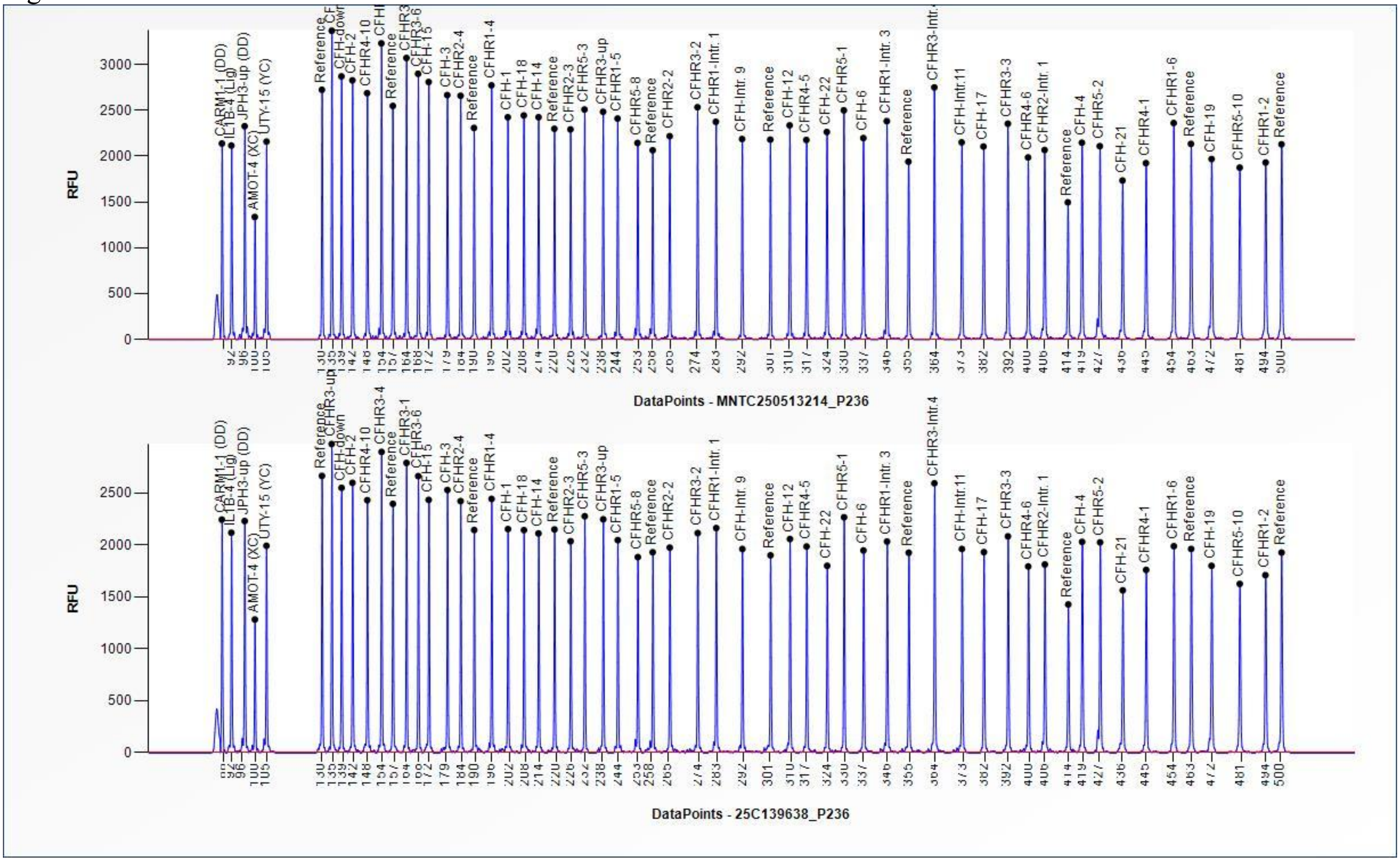

Beijing MyGenostics Medical Laboratory
Adress：No.9 Anqing Street, Juhong Building Block A, Airport Industrial Zone B, Shunyi District, Beijing City.
Tel.：010-87529591/400-003-5255.

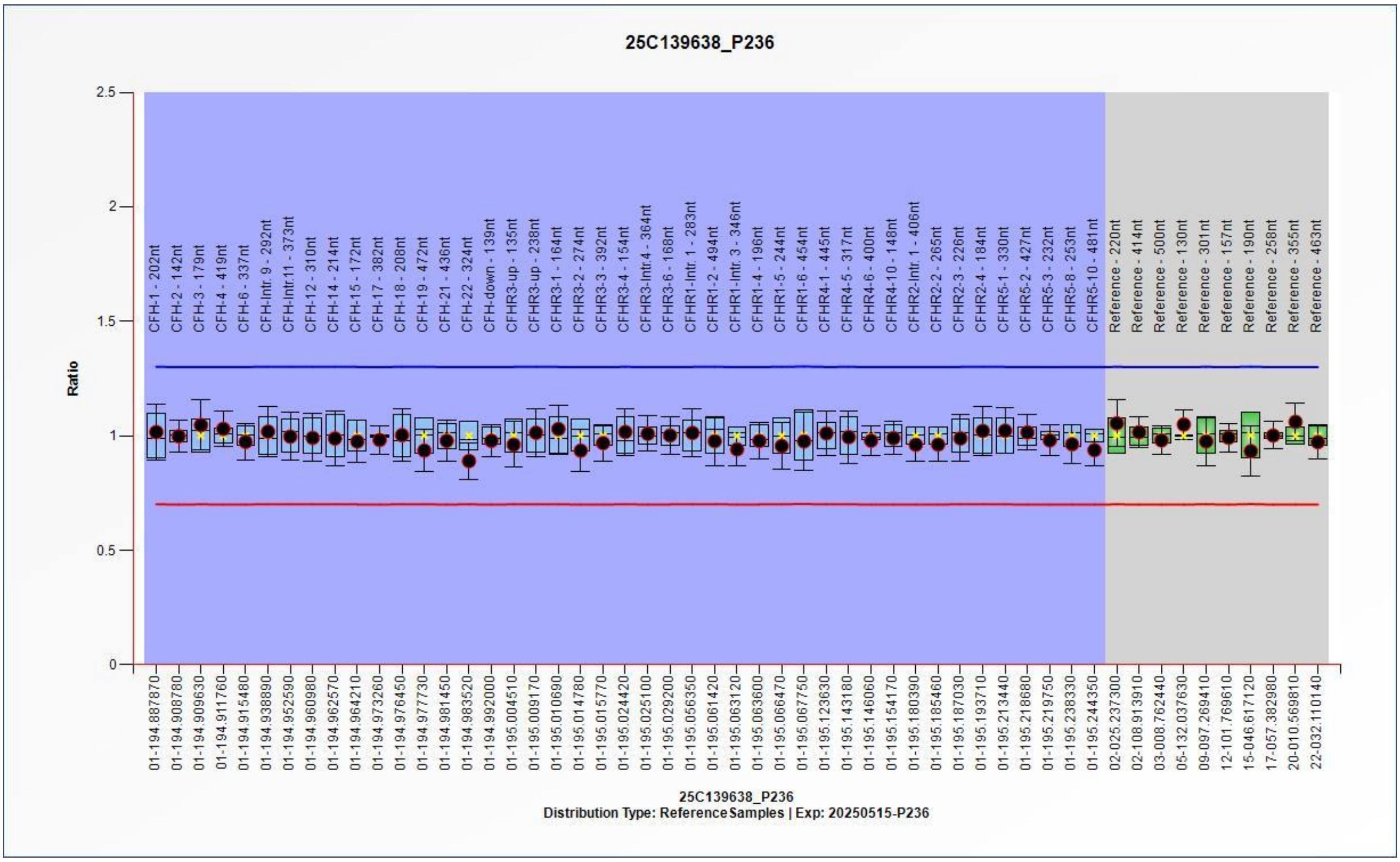

Analysis Note: A fluorescence signal intensity should be between 0.7 and 1.3

Disease Introduction/Genetic significance

Atypical hemolytic uremic syndrome (aHUS) is a clinical syndrome characterized primarily by the triad of microangiopathic hemolytic anemia, acute renal injury, and thrombocytopenia. Unlike typical HUS caused by Shiga toxin-producing Escherichia coli (STEC) infection, the primary pathogenic mechanism in aHUS patients involves abnormal activation of the complement bypass pathway. The incidence of aHUS is approximately 7/1000000. Congenital or acquired abnormal activation of complement pathway is the main pathogenic mechanism of aHUS. Most patients have gene mutations in complement-related factors (CFH, CFI, and CFHR proteins), while some patients are triggered by infections, medications, autoimmune diseases, or inherited cobalamin C metabolic defects.

The CFH gene is a member of the complement activation regulator (RCA) gene cluster, encoding a protein with 20 short consensus repeat (SCR) domains. This protein is secreted into the bloodstream and plays a critical role in complement activation regulation. The complement factor H (CFH) gene family is located on chromosome 1q31.3 and comprises six genes: CFH, CFHR3, CFHR1, CFHR4, CFHR2, and CFHR5. Mutations in this gene are associated with atypical hemolytic uremic syndrome (aHUS) and chronic hypocomplementemic nephropathy. The pathogenic mechanisms primarily include: regulatory region disruption: affecting CFH expression or interactions with other complement factors; gene fusion: forming heterozygous deletions with adjacent genes (such as CFHR1), resulting in aberrant proteins (such as CFH-CFHR1 fusion proteins) that interfere with complement regulatory functions; linkage disequilibrium: linked to specific haplotypes (such as high-risk haplotypes for aHUS). Francis N J et al. found a deletion in the C-terminal region of the CFH gene in a large lineage of atypical hemolytic uremic syndrome (aHUS) in 2012.Microhomologous sequences flanking the breakpoint suggested that this deletion occurred via microhomology-mediated end joining (MMEJ) rather than non-allelic homologous recombination (NAHR). The deletion

led to the formation of a CFH/CFHR3 heterozygous gene, with the N-terminal exon derived from CFH and the C-terminal exon from CFHR3. Functional analysis revealed that although the heterozygous protein exhibits complement regulation activity in liquid phase, it demonstrates defective capacity for complement regulation at the cell surface. Blood. 2008 Feb 1;111(3):1512-4. Epub 2007 Nov15 reported that homozygous deletion of CFHR1/CFHR3 constitutes a risk factor for atypical hemolytic uremic syndrome (HUS). The study elucidated a critical mechanism underlying atypical HUS, namely the production of CFH autoantibodies. These findings suggest that CFHR1/CFHR3 deletion represents a risk factor for CFH autoantibody generation.

Detection range

Probemix	Deletions/Duplications	Point mutation	Methylation	Gene/Region	Exon
P236	Yes	No	No	*CFH*	exon1-4，6，12，14，15，17，18，19，21，22，Intron 9，Intron 11，8.8 kb after exon 22
				CFHR1	exon4-6，2，Intron 1，Intron 3
				CFHR2	exon2，3，4，Intron 1
				CFHR3	exon1-4，6，Upstream，Intron 4
				CFHR4	exon1，5，6，10
				CFHR5	exon1，2，3，8，10

Detection methods and limitations

1. This test utilizes Multiplex Ligation-dependent Probe Amplification (MLPA) technology to analyze the relevant genes.
2. This test cannot detect any changes that lie outside the target sequence of the probes and will not detect copy number neutral inversions or translocations.
3. This test cannot distinguish between triploid and diploid cells in female samples. Furthermore, mRNA quantification requires specially selected samples and is subject to limitations in kinetic curve analysis
4. The interpretation of results at the borderline (critical) value requires further evaluation in conjunction with the clinical context.

Test member: Qing Zhang

Person Approving: 李淑葵

Analyst: Jing Yang

Report Date: 2025-05-19

*This report is issued by the entrusted party and/or its affiliated laboratory

*This test is only responsible for the tested sample.

*All the results above are lab data, for Clinical reference only. Furthermore, any genetic testing carries a very small but inherent risk of error.

*The content of the report may not be reproduced, disseminated or excerpted for publication without authorization.

*MyGenostics.Inc owns the final interpretation, if you have any doubts about the report, please contacts us within 5 days.

Beijing MyGenostics Medical Laboratory
Adress：No.9 Anqing Street, Juhong Building Block A, Airport Industrial Zone B, Shunyi District, Beijing City.
Tel.：010-87529591/400-003-5255.
