# Supplementary material for: Atypical hemolytic uremic syndrome in a patient with thalassemia and a CFH gene mutation: a case report
Source: Front Med (Lausanne). 2026 Apr 27;13:1659141. doi: 10.3389/fmed.2026.1659141 (PMC13159203; doi:10.3389/fmed.2026.1659141)
Supplement: Supplementary file 2 [file Data_Sheet_2.pdf]

Molecular genetic testing report

金域医学 KingMed Diagnostics

Name	Xu Yao	Specimen type	Blood	Medical Record No.	
Gender	Female	Barcode	2433175842	Bed No.	
Age	30Y	Test No.	NP26S07725	Specimen collected	
Hospital	GUIZHOU PROVINCIAL PEOPLE'S HOSPITAL			Specimen received	2024-07-25
Division	Nephrological Dept.			Report Date	2024-08-15
Physician				Report template	V1.6

Test performed：aHUS/TMA/C3GP Related Genes Panel

- Detection technology: Capture and sequence the target regions of aHUS/TMA/C3GP related genes (see attached table) on the genomic DNA of the subject.
- Analysis content: Based on the second-generation sequencing data, the single nucleotide variation, small indels and copy number variants of parts of aHUS/TMA/C3GP related genes were analyzed.

Diagnosis：

After 10+ days of elevated creatinine, a complete examination was conducted to consider atypical hemolytic uremia with acute kidney injury. The mother has thalassemia.

Result： **Postive. One likely pathogenic variant detected.**

Table One：

The clinical manifestations and expected genetic patterns of diseases caused by variation in this table are consistent with the main clinical phenotypes and zygote types of the subjects, respectively, and the variation rating is suspected to be pathogenic or pathogenic. It is suggested that clinicians should pay close attention to the clinical practice and carry out disease management, genetic counseling, and reproductive risk assessment/control.

- 1.1 SNV/Indel

Gene	Chromosome coordinates	Nomenclature	Zygosity	Disease	Origin	Variant Classification
			NA			

GZ003TA3PILCPZB

线路信息:GY贵阳-VIP周日一线

- ## 1.2 Copy number variant（CNV）

Chromosome coordinates	Variant Type	Genes	Disease	Origin	Variant Classification
chr1q31.3	Heterozygous deletion	*CFH*	HEMOLYTIC UREMIC SYNDROME, ATYPICAL, SUSCEPTIBILITY TO, 1[MIM:235400]; COMPLEMENT FACTOR H DEFICIENCY[MIM:609814]	NA	Likely pathogenic

*Due to the limitations of CNV analysis based on NGS data, the accuracy of this signal cannot be determined at present. This result is for the clinician's reference.

wes[hg19]chr1q31.3(196705953-196712758)X1 6.8Kb deletion

Analysis of the sequencing data from this patient indicates a heterozygous copy number deletion of approximately 6.8 kb in the chromosome 1q31.3 region of the patient’ s genomic DNA. This deletion may involve exons 16 - 20 and the adjacent introns of the CFH gene [NM_000186.4]. Pathogenic variants in the CFH gene can cause atypical hemolytic uremic syndrome type 1 (Hemolytic Uremic Syndrome, Atypical, Susceptibility to, 1; AHUS1) [MIM:235400] and complement factor H deficiency (CFHD) [MIM:609814]. The inheritance pattern is mainly autosomal dominant, although autosomal recessive inheritance has also been reported in the literature. HUS is characterized by hemolytic anemia, thrombocytopenia, and renal failure caused by microvascular platelet thrombi in the kidneys and other organs. AHUS can manifest from the neonatal period to adulthood. Hereditary AHUS accounts for approximately 60% of all AHUS cases. Even after complete recovery, patients with hereditary AHUS may experience frequent relapses, and 60% of hereditary AHUS cases progress to end-stage renal disease. Incomplete penetrance has been observed in this condition. Complement factor H deficiency, also known as C3 glomerulopathy, is a rare complement-mediated kidney disease caused by abnormal activation of the alternative complement pathway in the blood. The main manifestations include hematuria, proteinuria, acute nephritic syndrome or nephrotic syndrome, and decreased complement C3 levels. Approximately half of the patients eventually develop end-stage renal disease, and some patients experience vision loss. Incomplete penetrance has also been reported for this

condition. No similar cases involving the copy number deletion region identified in this patient have been reported in the DECIPHER database, and no literature reports are available in the HGMD database. Based on current evidence, this deletion variant is classified as a likely pathogenic variant.

It should be noted that this analysis is based on large fragment copy number variation detection using next-generation sequencing data; the accuracy and extent of the large genomic fragment change have not been confirmed by independent copy number analysis experiments. Therefore, this result is for clinical reference only and requires experimental validation using copy number detection methods (e.g., MLPA, CNV array) before it can be used for diagnosis or clinical decision-making. It is recommended that the clinician perform further CNV confirmation and analysis on the patient and her family members, and also conduct comprehensive clinical examinations on the patient based on the disease information described above to further interpret this result. Genetic counseling is recommended for this family.

Table Two：

The main clinical manifestations of the diseases associated with these mutations in this table correspond to the subject’ s primary clinical phenotype. However, the mutation is classified as having unclear clinical significance, or the expected genetic pattern of the disease does not match the detected genotype. This table also includes mutations where the pathogenicity is well-defined, the expected genetic pattern matches the genotype, but the disease exhibits a low expressivity or significant individual differences in clinical symptoms.

- 2.1 SNV/Indel

Gene	Chromosome coordinates	Nomenclature	Zygosity	Disease	Origin	Variant Classification

NA

Molecular genetic testing report

Name	Xu Yao	Specimen type	Blood	Medical Record No.	
Gender	Female	Barcode	2433175842	Bed No.	
Age	30Y	Test No.	NP26S07725	Specimen collected	
Hospital	GUIZHOU PROVINCIAL PEOPLE'S HOSPITAL			Specimen received	2024-07-25
Division	Nephrological Dept.			Report Date	2024-08-15
Physician				Report template	V1.6

- 2.2 Copy number variant（CNV）

Chromosome coordinates	Variant Type	Genes	Disease	Origin	Variant Classification
NA					

Table Three：Other variations related only to certain clinical phenotypes of the subject：

The findings listed in the table below are associated with certain clinical phenotypes of the subject. Currently, it is not possible to fully explain the relationship between these findings and the subject’ s phenotypes. This information is provided for clinicians’ reference. Examples include: 1) The expected genetic pattern of the disease does not match the detected homozygous genotype, or there is no complete genotype-phenotype correspondence within the family; 2) The main clinical manifestations of the disease do not correspond well with the subject’ s actual symptoms. For diseases caused by autosomal recessive inheritance, if a single heterozygous SNV/Indel variation with unclear clinical significance is found in a gene, such variations will not be included in the official report but will be listed in the extended dataset.

Gene	Chromosome coordinates	Nomenclature	Zygosity	Disease	Origin	Variant Classification
NA						

Principal Testing Laboratory: Guangzhou KingMed; National Customer Service Hotline: 4001-111-120; Address: No. 10, Luoxuan San Road, Guangzhou International Bio Island, Guangzhou, China

TEST PERFORMED 汪梦月 RESULT CHECKED [signature] REPORT SIGNED [signature]

Declaration

This report is an English translation based on the original report. All contents are subject to the original report.

Test Method And Limitations

1 . This test applied capture-based high-throughput sequencing technology to sequence the genes listed in test scope. The average sequencing depth on the exons of the genes contained in this test and their upstream and downstream 5bp were ≥90X, and the propotion of target regions with sequencing depth greater than 20X was about 98%. This method can not completely cover repetitive regions and GC-rich regions, and is not suitable for gene dynamic variation, complex recombination, variation in gene regions with highly homologous sequences on the genome, and variation in the deep part of gene regulatory region and intron region.

2 . The testing methodology employed has demonstrated a sensitivity of 99% for detecting copy number variations (CNVs) larger than 100 kb during internal validation. The detection rate for CNVs smaller than 100 kb depends on the genomic architecture and the sequencing coverage of the relevant regions. The accuracy and precise boundaries of any copy number variations identified through this analysis have not been confirmed by an independent, dedicated CNV detection assay. Therefore, these findings are provided for the clinician's reference and must be validated by a confirmatory test specifically designed for copy number analysis before they can be used for diagnosis or to guide clinical decision-making.

3 . This test will not report all the identified variants, but only report those variants with enough evidence in the related genes that can or may interpret the proband' s phenotype. Variants reported using HGVS nomenclature. The single nucleotide variation and small insertions / deletions and CNVs in the report were classified according to the standards and guidelines for the interpretation of sequence variation issued by the American Society of Medical Genetics and Genomics (ACMG) and the American Association for Molecular Pathology (AMP) or The Clinical Genome Resource (ClinGen).

Test Scope

• GENE LIST

ADAMTS13	*C3*	*CD46*	*CD59*	*CFB*	*CFH*
CFI	*DGKE*	*LCAT*	*MMACHC*	*MMADHC*	*MMUT*
MTR	*MTRR*	*PLG*	*SOX18*	*THBD*	

Reference

1. Noris M, Bresin E, Mele C, et al. Genetic Atypical Hemolytic-Uremic Syndrome. 2007 Nov 16[Updated 2021 Sep 23]. In: Adam MP, Everman DB, Mirzaa GM, et al., editors. GeneReviews.[Internet]. Seattle (WA): University of Washington, Seattle; 1993-2023.

2. Martín B, Smith RJH. C3 Glomerulopathy. 2007 Jul 20 [Updated 2018 Apr 5]. In: Adam MP, Everman DB, Mirzaa GM, et al., editors. GeneReviews.[Internet]. Seattle (WA): University of Washington, Seattle; 1993-2023.

GZ003TA3PILCPZB
线路信息:GY贵阳-VIP周日一线

3. Groopman EE, Marasa M, Cameron-Christie S, et al. Diagnostic Utility of Exome Sequencing for Kidney Disease. N Engl J Med, 2019, 380(2):142-151.
4. Karczewski K J, Francioli L C, Tiao G, et al. The mutational constraint spectrum quantified from variation in 141,456 humans[J]. Nature, 2020, 581(7809): 434-443.
5. Richards S, Aziz N, Bale S, et al. Standards and guidelines for the interpretation of sequence variants: a joint consensus recommendation of the American College of Medical Genetics and Genomics and the Association for Molecular Pathology[J]. Genetics in medicine, 2015, 17(5): 405-423.
6. Riggs E R, Andersen E F, Cherry A M, et al. Technical standards for the interpretation and reporting of constitutional copy-number variants: a joint consensus recommendation of the American College of Medical Genetics and Genomics (ACMG) and the Clinical Genome Resource (ClinGen)[J]. Genetics in Medicine, 2020, 22(2): 245-257.
7. Stenson P D, Mort M, Ball E V, et al. The Human Gene Mutation Database (HGMD: optimizing its use in a clinical diagnostic or research setting[J]. Human genetics, 2020, 139(10): 1197-1207.

GZ003TA3PILCPZB
线路信息:GY贵阳-VIP周日一线

金域医学 KingMed Diagnostics

广州金域医学检验中心

GUANGZHOU KINGMED CENTER FOR CLINICAL LABORATORY

Molecular genetic testing report

Barcode	2433175842	Referring hospital	GUIZHOU PROVINCIAL PEOPLE'S HOSPITAL		
Name	Xu Yao	Division	Nephrological Dept.	Test No.	QP23D0039
Gender	Female	Bed No.		Medical Record No.	
Age	30Y	Specimen received	2025-03-18 14:12:46	Referring Physician	
Specimen type	Blood	Specimen collected		Phone No.	
Specimen state	No visible abnormality				
Diagnosis:	After 10+ days of elevated creatinine, a complete examination was conducted to consider atypical hemolytic uremia with acute kidney injury. The mother has thalassemia.				

Test performed:
Semi-quantitative PCR verification of specific gene deletion mutations

Results and Interpretations

Xu Yao（QP23D0039）（Original experiment number：NP26S07725）： [hg19]chr1q31.3 (196705953-196712758)X1，deletion;

Xu Yao’s father Xu Benyong（VP27D02013）： [hg19]chr1q31.3 (196705953-196712758)X2，normal;

Xu Yao’s mother Peng Mei（VP27D02014）： [hg19]chr1q31.3 (196705953-196712758)X2，normal;

1. Xu Yao (QP23D0039) (Original experiment number: NP26S07725)

High-throughput sequencing results indicated a copy number deletion of approximately 6.8 kb in the region chr1:196705953-196712758. In this experiment, Xu Yao showed a deletion in this region, while her father Xu Benyong and her mother Peng Mei showed normal copy numbers in this region. It is speculated that the deletion in Xu Yao is a de novo variant.

2. The results of this experiment support the copy number variation detected by high-throughput sequencing in the proband.

3.Analysis of the sequencing data from this patient indicates a heterozygous copy number deletion of approximately 6.8 kb in the chromosome 1q31.3 region of the patient’s genomic DNA. This deletion may involve exons 16–20 and the adjacent introns of the CFH gene [NM_000186.4]. Pathogenic variants in the CFH gene can cause atypical hemolytic uremic syndrome type 1 (Hemolytic Uremic Syndrome, Atypical, Susceptibility to, 1; AHUS1) [MIM:235400] and complement factor H deficiency (CFHD) [MIM:609814]. The inheritance pattern is mainly autosomal dominant, although autosomal recessive inheritance has also been reported in the literature. HUS is characterized by hemolytic anemia, thrombocytopenia, and renal failure caused by microvascular platelet thrombi in the kidneys and other organs. AHUS can manifest from the neonatal period to adulthood. Hereditary AHUS accounts for approximately 60% of all AHUS cases. Even after complete recovery, patients with hereditary AHUS may experience frequent relapses, and 60% of hereditary AHUS cases progress to end-stage renal disease. Incomplete penetrance has been observed in this condition. Complement factor H deficiency, also known as C3 glomerulopathy, is a rare complement-mediated kidney disease caused by abnormal activation of the alternative complement pathway in the blood. The main manifestations include hematuria, proteinuria, acute nephritic syndrome or

GZ003TA3PILCPZB
线路信息:GY贵阳-VIP周日一线

nephrotic syndrome, and decreased complement C3 levels. Approximately half of the patients eventually develop end-stage renal disease, and some patients experience vision loss. Incomplete penetrance has also been reported for this condition. No similar cases involving the copy number deletion region identified in this patient have been reported in the DECIPHER database, and no literature reports are available in the HGMD database. Based on current evidence, this deletion variant is classified as a likely pathogenic variant.

Test Method

1. This test uses semi-quantitative PCR technology to detect whether there are copy number variations of specific genes in the samples to be tested.
2. This test is mainly used for confirmation of results suspected of having specific gene deletion or duplication variants.

Reference

PLoS One. 2014 Jan 31 ; 9 (1) : e87472. doi : 10.1371/journal.pone.0087472. eCollection 2014.

Declaration

1. It is recommended to seek genetic counseling at an institution with genetic counseling qualifications.
2. This laboratory is accredited by CAP(College of American Pathologists).
3. This report is an English translation based on the original report. All contents are subject to the original report.

**TEST PERFORMED& RESULT CHECKED**	何恺丰	**REPORT SIGNED:**	邓安[illegible]

Principal Testing Laboratory: Guangzhou KingMed

Address: No. 10, Luoxuan San Road, Guangzhou International Bio Island, Guangzhou, China

National Customer Service Hotline: 4001-111-120

Website: www.kingmed.com.cn

Report Date: 2025-03-25 17:59:32

GZ003TA3PILCPZB
线路信息:GY贵阳-VIP周日一线

Molecular genetic testing report

金域医学 KingMed Diagnostics

Name	Xu Yao	Specimen type	Blood	Medical Record No.	
Gender	Female	Barcode	2433175842	Bed No.	
Age	30Y	Test No.	NP26S07725	Specimen collected	
Hospital	GUIZHOU PROVINCIAL PEOPLE'S HOSPITAL			Specimen received	2024-07-25
Division	Nephrological Dept.			Report Date	2024-08-15
Physician				Report template	V1.6

Test performed：aHUS/TMA/C3GP Related Genes Panel

- Detection technology: Capture and sequence the target regions of aHUS/TMA/C3GP related genes (see attached table) on the genomic DNA of the subject.
- Analysis content: Based on the second-generation sequencing data, the single nucleotide variation, small indels and copy number variants of parts of aHUS/TMA/C3GP related genes were analyzed.

Diagnosis：

After 10+ days of elevated creatinine, a complete examination was conducted to consider atypical hemolytic uremia with acute kidney injury. The mother has thalassemia.

Result： Postive. One likely pathogenic variant detected.

Table One：

The clinical manifestations and expected genetic patterns of diseases caused by variation in this table are consistent with the main clinical phenotypes and zygote types of the subjects, respectively, and the variation rating is suspected to be pathogenic or pathogenic. It is suggested that clinicians should pay close attention to the clinical practice and carry out disease management, genetic counseling, and reproductive risk assessment/control.

- 1.1 SNV/Indel

Gene	Chromosome coordinates	Nomenclature	Zygosity	Disease	Origin	Variant Classification
			NA			

- ## 1.2 Copy number variant（CNV）

Chromosome coordinates	Variant Type	Genes	Disease	Origin	Variant Classification
chr1q31.3	Heterozygous deletion	*CFH*	HEMOLYTIC UREMIC SYNDROME, ATYPICAL, SUSCEPTIBILITY TO, 1[MIM:235400]; COMPLEMENT FACTOR H DEFICIENCY[MIM:609814]	NA	Likely pathogenic

*Due to the limitations of CNV analysis based on NGS data, the accuracy of this signal cannot be determined at present. This result is for the clinician's reference.

wes[hg19]chr1q31.3(196705953-196712758)X1   6.8Kb   deletion

Analysis of the sequencing data from this patient indicates a heterozygous copy number deletion of approximately 6.8 kb in the chromosome 1q31.3 region of the patient’s genomic DNA. This deletion may involve exons 16 - 20 and the adjacent introns of the CFH gene [NM_000186.4]. Pathogenic variants in the CFH gene can cause atypical hemolytic uremic syndrome type 1 (Hemolytic Uremic Syndrome, Atypical, Susceptibility to, 1; AHUS1) [MIM:235400] and complement factor H deficiency (CFHD) [MIM:609814]. The inheritance pattern is mainly autosomal dominant, although autosomal recessive inheritance has also been reported in the literature. HUS is characterized by hemolytic anemia, thrombocytopenia, and renal failure caused by microvascular platelet thrombi in the kidneys and other organs. AHUS can manifest from the neonatal period to adulthood. Hereditary AHUS accounts for approximately 60% of all AHUS cases. Even after complete recovery, patients with hereditary AHUS may experience frequent relapses, and 60% of hereditary AHUS cases progress to end-stage renal disease. Incomplete penetrance has been observed in this condition. Complement factor H deficiency, also known as C3 glomerulopathy, is a rare complement-mediated kidney disease caused by abnormal activation of the alternative complement pathway in the blood. The main manifestations include hematuria, proteinuria, acute nephritic syndrome or nephrotic syndrome, and decreased complement C3 levels. Approximately half of the patients eventually develop end-stage renal disease, and some patients experience vision loss. Incomplete penetrance has also been reported for this

Name	Xu Yao	Specimen type	Blood	Medical Record No.	
Gender	Female	Barcode	2433175842	Bed No.	
Age	30Y	Test No.	NP26S07725	Specimen collected	
Hospital	GUIZHOU PROVINCIAL PEOPLE'S HOSPITAL			Specimen received	2024-07-25
Division	Nephrological Dept.			Report Date	2024-08-15
Physician				Report template	V1.6

condition. No similar cases involving the copy number deletion region identified in this patient have been reported in the DECIPHER database, and no literature reports are available in the HGMD database. Based on current evidence, this deletion variant is classified as a likely pathogenic variant.

It should be noted that this analysis is based on large fragment copy number variation detection using next-generation sequencing data; the accuracy and extent of the large genomic fragment change have not been confirmed by independent copy number analysis experiments. Therefore, this result is for clinical reference only and requires experimental validation using copy number detection methods (e.g., MLPA, CNV array) before it can be used for diagnosis or clinical decision-making. It is recommended that the clinician perform further CNV confirmation and analysis on the patient and her family members, and also conduct comprehensive clinical examinations on the patient based on the disease information described above to further interpret this result. Genetic counseling is recommended for this family.

Table Two：

The main clinical manifestations of the diseases associated with these mutations in this table correspond to the subject’ s primary clinical phenotype. However, the mutation is classified as having unclear clinical significance, or the expected genetic pattern of the disease does not match the detected genotype. This table also includes mutations where the pathogenicity is well-defined, the expected genetic pattern matches the genotype, but the disease exhibits a low expressivity or significant individual differences in clinical symptoms.

- 2.1 SNV/Indel

Gene	Chromosome coordinates	Nomenclature	Zygosity	Disease	Origin	Variant Classification
			NA			

Name	Xu Yao	Specimen type	Blood	Medical Record No.	
Gender	Female	Barcode	2433175842	Bed No.	
Age	30Y	Test No.	NP26S07725	Specimen collected	
Hospital	GUIZHOU PROVINCIAL PEOPLE'S HOSPITAL			Specimen received	2024-07-25
Division	Nephrological Dept.			Report Date	2024-08-15
Physician				Report template	V1.6

- 2.2 Copy number variant（CNV）

Chromosome coordinates	Variant Type	Genes	Disease	Origin	Variant Classification
NA					

Table Three：Other variations related only to certain clinical phenotypes of the subject：

The findings listed in the table below are associated with certain clinical phenotypes of the subject. Currently, it is not possible to fully explain the relationship between these findings and the subject’ s phenotypes. This information is provided for clinicians’ reference. Examples include: 1) The expected genetic pattern of the disease does not match the detected homozygous genotype, or there is no complete genotype-phenotype correspondence within the family; 2) The main clinical manifestations of the disease do not correspond well with the subject’ s actual symptoms. For diseases caused by autosomal recessive inheritance, if a single heterozygous SNV/Indel variation with unclear clinical significance is found in a gene, such variations will not be included in the official report but will be listed in the extended dataset.

Gene	Chromosome coordinates	Nomenclature	Zygosity	Disease	Origin	Variant Classification
NA						

Principal Testing Laboratory: Guangzhou KingMed; National Customer Service Hotline: 4001-111-120; Address: No. 10, Luoxuan San Road, Guangzhou International Bio Island, Guangzhou, China

TEST PERFORMED 汪梦月 RESULT CHECKED REPORT SIGNED

Declaration

This report is an English translation based on the original report. All contents are subject to the original report.

Test Method And Limitations

1． This test applied capture-based high-throughput sequencing technology to sequence the genes listed in test scope. The average sequencing depth on the exons of the genes contained in this test and their upstream and downstream 5bp were ≥90X, and the propotion of target regions with sequencing depth greater than 20X was about 98%. This method can not completely cover repetitive regions and GC-rich regions, and is not suitable for gene dynamic variation, complex recombination, variation in gene regions with highly homologous sequences on the genome, and variation in the deep part of gene regulatory region and intron region.

2． The testing methodology employed has demonstrated a sensitivity of 99% for detecting copy number variations (CNVs) larger than 100 kb during internal validation. The detection rate for CNVs smaller than 100 kb depends on the genomic architecture and the sequencing coverage of the relevant regions. The accuracy and precise boundaries of any copy number variations identified through this analysis have not been confirmed by an independent, dedicated CNV detection assay. Therefore, these findings are provided for the clinician's reference and must be validated by a confirmatory test specifically designed for copy number analysis before they can be used for diagnosis or to guide clinical decision-making.

3． This test will not report all the identified variants, but only report those variants with enough evidence in the related genes that can or may interpret the proband’ s phenotype. Variants reported using HGVS nomenclature. The single nucleotide variation and small insertions / deletions and CNVs in the report were classified according to the standards and guidelines for the interpretation of sequence variation issued by the American Society of Medical Genetics and Genomics (ACMG) and the American Association for Molecular Pathology (AMP) or The Clinical Genome Resource (ClinGen).

Test Scope

• GENE LIST

ADAMTS13	*C3*	*CD46*	*CD59*	*CFB*	*CFH*
CFI	*DGKE*	*LCAT*	*MMACHC*	*MMADHC*	*MMUT*
MTR	*MTRR*	*PLG*	*SOX18*	*THBD*	

Reference

1. Noris M, Bresin E, Mele C, et al. Genetic Atypical Hemolytic-Uremic Syndrome. 2007 Nov 16[Updated 2021 Sep 23]. In: Adam MP, Everman DB, Mirzaa GM, et al., editors. GeneReviews.[Internet]. Seattle (WA): University of Washington, Seattle; 1993-2023.

2. Martín B, Smith RJH. C3 Glomerulopathy. 2007 Jul 20 [Updated 2018 Apr 5]. In: Adam MP, Everman DB, Mirzaa GM, et al., editors. GeneReviews.[Internet]. Seattle (WA): University of Washington, Seattle; 1993-2023.

GZ003TA3PILCPZB
线路信息:GY贵阳-VIP周日一线

3. Groopman EE, Marasa M, Cameron-Christie S, et al. Diagnostic Utility of Exome Sequencing for Kidney Disease. N Engl J Med, 2019, 380(2):142-151.
4. Karczewski K J, Francioli L C, Tiao G, et al. The mutational constraint spectrum quantified from variation in 141,456 humans[J]. Nature, 2020, 581(7809): 434-443.
5. Richards S, Aziz N, Bale S, et al. Standards and guidelines for the interpretation of sequence variants: a joint consensus recommendation of the American College of Medical Genetics and Genomics and the Association for Molecular Pathology[J]. Genetics in medicine, 2015, 17(5): 405-423.
6. Riggs E R, Andersen E F, Cherry A M, et al. Technical standards for the interpretation and reporting of constitutional copy-number variants: a joint consensus recommendation of the American College of Medical Genetics and Genomics (ACMG) and the Clinical Genome Resource (ClinGen)[J]. Genetics in Medicine, 2020, 22(2): 245-257.
7. Stenson P D, Mort M, Ball E V, et al. The Human Gene Mutation Database (HGMD: optimizing its use in a clinical diagnostic or research setting[J]. Human genetics, 2020, 139(10): 1197-1207.

广州金域医学检验中心

GUANGZHOU KINGMED CENTER FOR CLINICAL LABORATORY

Molecular genetic testing report

Barcode	2433175842	Referring hospital	GUIZHOU PROVINCIAL PEOPLE'S HOSPITAL		
Name	Xu Yao	Division	Nephrological Dept.	Test No.	QP23D0039
Gender	Female	Bed No.		Medical Record No.	
Age	30Y	Specimen received	2025-03-18 14:12:46	Referring Physician	
Specimen type	Blood	Specimen collected		Phone No.	
Specimen state	No visible abnormality				
Diagnosis:	After 10+ days of elevated creatinine, a complete examination was conducted to consider atypical hemolytic uremia with acute kidney injury. The mother has thalassemia.				

Test performed:
Semi-quantitative PCR verification of specific gene deletion mutations

Results and Interpretations

Xu Yao（QP23D0039）（Original experiment number：NP26S07725）： [hg19]chr1q31.3 (196705953-196712758)X1，deletion;

Xu Yao’s father Xu Benyong（VP27D02013）： [hg19]chr1q31.3 (196705953-196712758)X2，normal;

Xu Yao’s mother Peng Mei（VP27D02014）： [hg19]chr1q31.3 (196705953-196712758)X2，normal;

1. Xu Yao (QP23D0039) (Original experiment number: NP26S07725)

High-throughput sequencing results indicated a copy number deletion of approximately 6.8 kb in the region chr1:196705953-196712758. In this experiment, Xu Yao showed a deletion in this region, while her father Xu Benyong and her mother Peng Mei showed normal copy numbers in this region. It is speculated that the deletion in Xu Yao is a de novo variant.

2. The results of this experiment support the copy number variation detected by high-throughput sequencing in the proband.

3.Analysis of the sequencing data from this patient indicates a heterozygous copy number deletion of approximately 6.8 kb in the chromosome 1q31.3 region of the patient’s genomic DNA. This deletion may involve exons 16–20 and the adjacent introns of the CFH gene [NM_000186.4]. Pathogenic variants in the CFH gene can cause atypical hemolytic uremic syndrome type 1 (Hemolytic Uremic Syndrome, Atypical, Susceptibility to, 1; AHUS1) [MIM:235400] and complement factor H deficiency (CFHD) [MIM:609814]. The inheritance pattern is mainly autosomal dominant, although autosomal recessive inheritance has also been reported in the literature. HUS is characterized by hemolytic anemia, thrombocytopenia, and renal failure caused by microvascular platelet thrombi in the kidneys and other organs. AHUS can manifest from the neonatal period to adulthood. Hereditary AHUS accounts for approximately 60% of all AHUS cases. Even after complete recovery, patients with hereditary AHUS may experience frequent relapses, and 60% of hereditary AHUS cases progress to end-stage renal disease. Incomplete penetrance has been observed in this condition. Complement factor H deficiency, also known as C3 glomerulopathy, is a rare complement-mediated kidney disease caused by abnormal activation of the alternative complement pathway in the blood. The main manifestations include hematuria, proteinuria, acute nephritic syndrome or

GZ003TA3PILCPZB
线路信息:GY贵阳-VIP周日一线

nephrotic syndrome, and decreased complement C3 levels. Approximately half of the patients eventually develop end-stage renal disease, and some patients experience vision loss. Incomplete penetrance has also been reported for this condition. No similar cases involving the copy number deletion region identified in this patient have been reported in the DECIPHER database, and no literature reports are available in the HGMD database. Based on current evidence, this deletion variant is classified as a likely pathogenic variant.

Test Method

1. This test uses semi-quantitative PCR technology to detect whether there are copy number variations of specific genes in the samples to be tested.
2. This test is mainly used for confirmation of results suspected of having specific gene deletion or duplication variants.

Reference

PLoS One. 2014 Jan 31 ; 9 (1) : e87472. doi : 10.1371/journal.pone.0087472. eCollection 2014.

Declaration

1. It is recommended to seek genetic counseling at an institution with genetic counseling qualifications.
2. This laboratory is accredited by CAP(College of American Pathologists).
3. This report is an English translation based on the original report. All contents are subject to the original report.

TEST PERFORMED& RESULT CHECKED	何恺丰	REPORT SIGNED:	邓安琪

Principal Testing Laboratory: Guangzhou KingMed

Address: No. 10, Luoxuan San Road, Guangzhou International Bio Island, Guangzhou, China

National Customer Service Hotline: 4001-111-120

Website: www.kingmed.com.cn

Report Date: 2025-03-25 17:59:32

GZ003TA3PILCPZB
线路信息:GY贵阳-VIP周日一线
