# Supplementary figures and images for: Atypical hemolytic uremic syndrome in a patient with thalassemia and a CFH gene mutation: a case report
Source: Front Med (Lausanne). 2026 Apr 27;13:1659141. doi: 10.3389/fmed.2026.1659141 (PMC13159203; doi:10.3389/fmed.2026.1659141)

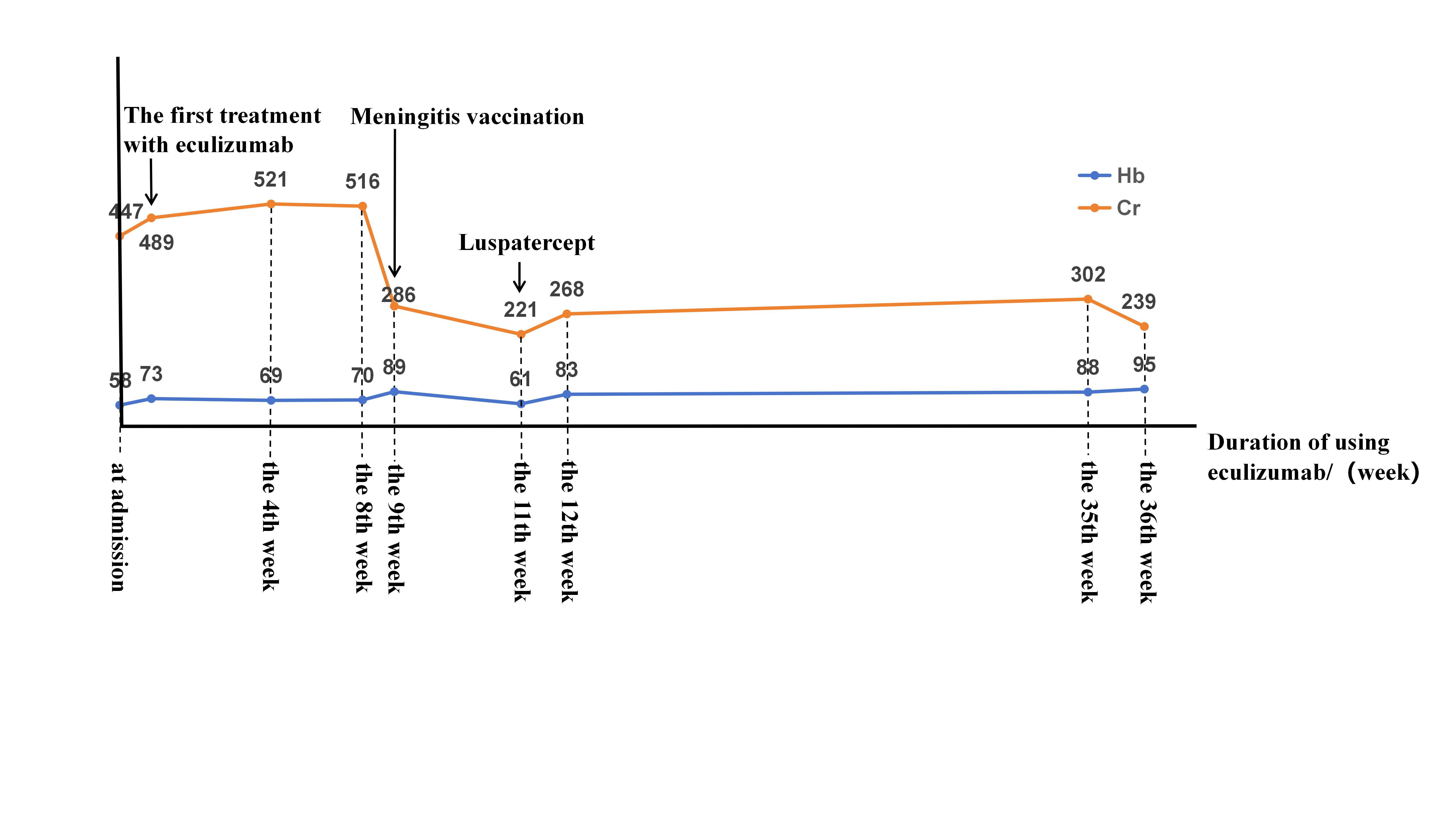

Supplement: Supplementary file 3 [file Image_1.jpeg]

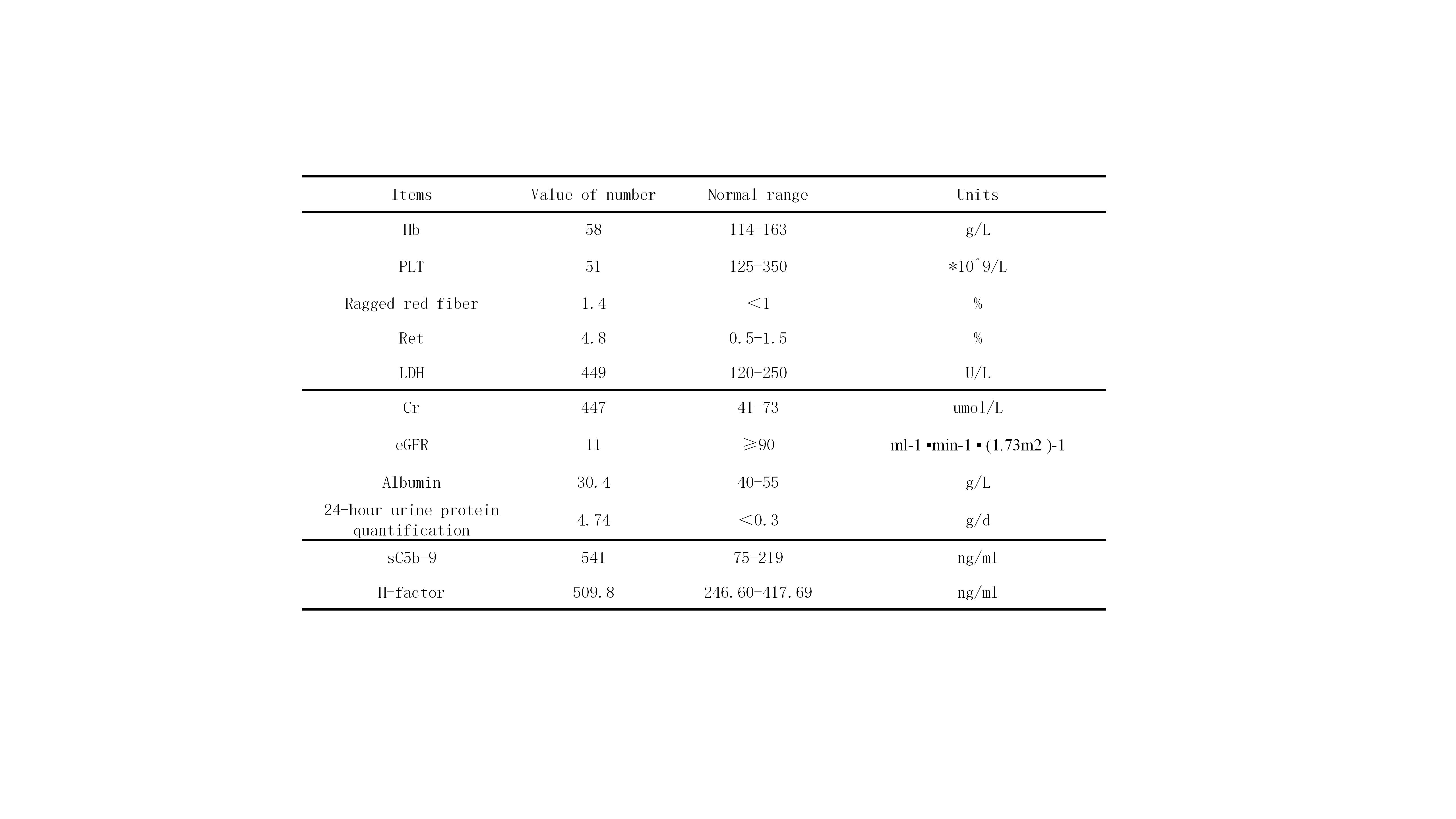

Supplement: Supplementary file 4 [file Image_2.jpeg]
